# Functional analysis of the human miRNome in non-small cell lung cancer unveils a novel miR-92b-3p/NOTCH3 axis that drives tumor progression

**DOI:** 10.1038/s41419-026-08709-x

**Published:** 2026-04-08

**Authors:** Roberto Cuttano, Miriam Kuku Afanga, Francesca Longo, Nicole Di Cillo, Valentina Melocchi, Elisa Dama, Francesco Mazzarelli, Pietro Palumbo, Giovanni Bertalot, Fabrizio Bianchi

**Affiliations:** 1 Unit of Cancer Biomarkers, Fondazione IRCCS Casa Sollievo della Sofferenza, San Giovanni Rotondo, Italy; 2 Unit of Medical Genetics, Fondazione IRCCS Casa Sollievo della Sofferenza, San Giovanni Rotondo, Italy; 3 Surgical Pathology, Santa Chiara Hospital-APSS, Trento, Italy; 4 Centre for Medical Sciences-CISMed, University of Trento, Trento, Italy

**Keywords:** Non-small-cell lung cancer, miRNAs

## Abstract

Lung cancer remains the leading cause of cancer-related mortality worldwide. MicroRNAs, endogenous non-coding RNA of ~22 nucleotides, play key regulatory roles in lung cancer–related mechanisms and represent promising diagnostic and prognostic biomarkers, as well as potential therapeutic agents. However, a comprehensive functional screening of human miRNAs in lung cancer was missing. To tackle this, we performed a lentiviral-based miRNA screening (~2500 microRNAs) of lung adenocarcinoma (LUAD) i.e. the most frequent lung cancer subtype. We discovered and annotated miRNAs functionally associated with proliferation, migration, and invasion of LUAD cells. We then integrated our functional miRNA screening with transcriptomic and clinical data from a large LUAD patient cohort (TCGA-LUAD), along with spatial transcriptomics (ST) of *in-house* LUAD samples. This approach enabled us to identify phenotype-specific miRNA signatures and develop a 15-miRNA risk score predictive of poor overall survival (HR = 2.48, *p* < 0.0001). Among these, miR-92b-3p, a less studied member of the oncogenic miR-17~92 cluster, emerged as a critical driver of LUAD progression. Transcriptomics analysis revealed that miR-92b-3p overexpression increase NOTCH3 signaling activation. The inhibition of NOTCH3 signaling, either genetically or pharmacologically, abrogated the pro-migratory and invasive effects of miR-92b-3p. In summary, our study provides a systematic functional annotation of the human miRNome in LUAD and identifies a novel miR-92b-3p/NOTCH3 axis that contributes to LUAD progression.

## Introduction

Despite advancements in our comprehension of lung cancer (LC) biology [1], our knowledge of LC driver mechanisms is still limited. As matter of fact, a quarter of non-small cell lung tumors (NSCLC) does not have any identified cancer drivers [2]. Bulk and single-cell omics analyses revealed a high genetic/epigenetic heterogeneity in LC, which complicates the identification of mechanisms functionally involved in LC progression [3, 4]. As a result, prognosis of LC patients remains dismal, which poses LC as the leading cause of cancer-related deaths worldwide [5]. Recently, functional genomic screening using CRISPR showed promising results to identify gene functions involved in cancer progression, including LC [6, 7]. However, CRISPR screening typically perturbs only one gene per cell, limiting its ability to investigate more complex tumor progression mechanisms that depend on interactions among multiple genes or pathways.

To tackle this, we used a strategy to perturb multiple targets/pathways by leveraging a human shMIMIC lentiviral library to modulate the expression of ~2500 microRNAs, virtually the whole miRNome, in lung adenocarcinoma (LUAD) cells, i.e. the most frequent LC subtype. A single microRNA usually targets hundreds of coding transcripts, and thus it is capable to modulate multiple cancer pathways [8]. Surprisingly, a comprehensive functional annotation of miRNAs implicated in LC is currently lacking.

Here, we combined shMIMIC screening with miRNA-seq analysis of human LUAD samples including a large cohort of patients we meta-analyzed (i.e., the TCGA cohort, *N* = 510), and with Spatial Transcriptomics (ST) analysis, to systematically interrogate miRNAs and relative targets, which are functionally related to LC proliferation, migration, and invasion. Importantly, we identified miR-92b-3p, an understudied member of the NOTCH1/MYC-regulated miR-17~92 cluster, as a modulator of LC aggressiveness by inducing NOTCH3 expression, which has been reported to drive lung cancer progression [9–11]. MiR-92b-3p correlates with LC poor prognosis, is enriched in invasive LC cells, and promotes migration and invasion, highlighting its potential as biomarker of a possible therapeutic target.

## Methods

### Cell lines

A549, NCI-H1975, NCI- H838 and NCI-H1944 were obtained from ATCC and cultured in RPMI (Gibco) with 10% FBS and 1% penicillin/streptomycin. All cell lines were grown at 37 °C in a humidified incubator with 5% CO2 and routinely tested for Mycoplasma contamination using PCR. Genetic background information for the cell lines was retrieved using VIKTOR (Virtual International Knowledgebase for Thoracic Oncology Research) (https://www.iaslc.org/viktor) [12].

### Lentivirus transduction with shMIMIC lentiviral microRNA pooled libraries

Pooled shMIMIC library (Horizon Discovery Ltd) contains 1 pool of lentiviral SMARTvector constructs overexpressing all human mature microRNAs as annotated in miRBase database (v21). All constructs in this library are under the activity of mCMV constitutive promoter and carry the fluorescent reporter TurboGFP as well as the puromycin resistance gene. A549 cells were transduced at MOI = 0.2 (two biological replicates for each condition were performed) to produce a population of cells with one integration event per cell. Fold representation of each construct was equal to 500 and was maintained constant during cell splitting. Following lentiviral transduction, cells that successfully carried the constructs were selected with 3 μg/ml of puromycin (Gibco) for 6 days. Cells were then used to perform the screening as described below.

### Lentiviral shRNA-transduced cell library screening

#### Proliferation screening

A549 cells transduced with the library were maintained after selection in culture for two weeks after selection then lysed for genomic DNA extraction (PRO) or, alternatively, lysed immediately after selection with puromycin (CTRL).

#### Migration and invasion screening

transduced A549 cells underwent three consecutive rounds of trans-well migration (MIG) and invasion (INV), or kept in culture without selective pressure (CTRL).

### Genomic DNA extraction, barcode amplification, and next-generation sequencing

At the end of each screening condition, genomic DNA was isolated using the Blood & Cell Culture DNA Mini Kit (Qiagen) according to the manufacturer’s instructions. Pooled barcodes were amplified by PCR reactions using 825 ng of genomic DNA and Phusion Hot Start II DNA Polymerase (Thermo Fisher Scientific) and the Illumina-adapted SMARTvector Indexing PCR primer Kit A and B (Horizon Discovery Ltd., PRM7668, PRM10186) according to the manufacturer’s instructions. PCR was performed at 98 °C for 3 min followed by 23 cycles of 98 °C for 10 s, 60 °C for 15 s, 72 °C for 15 s, and 72 °C for 5 min. PCR products were purified using the QIAquick™ PCR Purification Kit (Qiagen) following the manufacturer’s protocol. DNA was quantified at Qubit 4 fluorometer (Thermo Fisher Scientific) using Qubit™ 1X dsDNA High Sensitivity (Thermo Fisher Scientific). DNA was analyzed with 4200 TapeStation System (Agilent) using D1000 ScreenTapes (Agilent) to confirm the size of the amplicons. NGS of amplified barcodes was performed on the Illumina NextSeq 500 using the SMARTvector Read 1 Sequencing Primer, following the manufacturer’s protocol. Briefly, barcoded DNA libraries were pooled in equimolar concentrations and loaded onto the flow cell at a concentration of 1.3 picomolar and sequenced with a single read, 75 cycles strategy (Illumina Inc.)

### Sequencing data analysis and “hit” selection

Sequencing data obtained from the pooled lentiviral screening were processed according to the manufacturer’s indication. Briefly, an index was generated using Bowtie2 [13] based on a reference FASTA file containing construct sequences included in the library. Reads from each sample were then aligned to this reference index using Bowtie2 with custom parameters (-p 4 -3 53), obtaining .sam files. The .sam files were then converted into count table files using a python script provided by manufacturer. To identify differentially integrated constructs, we applied two different approaches: i) DESeq2 [14]; ii) quantitative trait analysis (Q-trait), using BRB-ArrayTools [15]. Differential integration analysis was performed using an R script provided by the manufacturer to compare reference and treated samples. Significant hits were defined as constructs with an adjusted *p*-value < 0.1 and an absolute mean fold-change >1.2, with a concordant regulation in the replicates. For Q-trait analysis we used as input file the normalized counts obtained from DESeq2. Significant hits were defined at the nominal 0.01 level of the univariate test. Data can be found in Supplementary Table S1.

### Lentivirus transduction and cell infection

shMIMIC Lentiviral microRNAs for miR-92b-3p (GSH11926-246415221), miR-582-5p (GSH11926-246419874), miR-379-3p (GSH11926-246415108), miR-29b-2-5p (GSH11926-246410125) and non-targeting control (VSC11722) cloned into SMARTvector lentiviral backbone were purchased from Horizon Discovery Ltd. The lentiviral particles were produced in 293T cells using a three-plasmid transfection system mediated by Lipofectamine™ 3000 transfection reagent (Thermo Fisher Scientific). Lentivirus-containing supernatants were collected 48 and 72 h after transfection, passed through a 0.45 μm filter, and concentrated using PEG (SBI). Lentiviral infections were carried out in the presence of 8 μg/ml polybrene (Sigma-Aldrich) followed by selection with 3 μg/ml puromycin (Gibco) for 6 days.

### Cell proliferation assay

Cells were plated at density of 2300 cells/well in 96-well plates in triplicate in 90 μL of complete media. Cells were analyzed at the indicated time points by adding CyQUANT Cell Proliferation Assay Kit (Thermo Fisher Scientific) in a ratio of 1:10 directly in complete media. Fluorescence was measured at 480/528 nm using a Synergy HT (Biotek) microplate reader.

### Migration/invasion trans-well assay

Cell migration and invasion assays were performed using trans-well chambers with 8.0 µm pore polycarbonate membranes (Falcon). For the invasion assay, membranes were pre-coated with Matrigel Growth Factor Reduced (Corning, Lot 4132005) at a concentration of 150 µg/ml in a 24-well plate. After O.N. starvation in RPMI medium without FBS, cells were resuspended in RPMI medium without FBS and seeded at a density of 3 × 10⁴ cells per 24-well trans-well insert into the upper chamber. The lower chamber was filled with RPMI medium with 20% FBS to serve as chemoattractant. Following 16 h of incubation at 37 °C in a humidified incubator with 5% CO2, trans-wells were fixed using 4% PFA and cells nuclei stained using DAPI (Sigma-Aldrich). For the library screening, 1.3 × 10⁶ cells were seeded in 6-well trans-well inserts to maintain fold representation of the construct. For migration/invasion assay in the presence of DAPT, cells were pre-treated with 5 µM DAPT for 48 h and invasion/migration assay was carried out in the presence of 5 µM DAPT. For migration/invasion assay in the presence of siRNA, cells were first pre-transfected with 10 nM of a siRNA against NOTCH3 or a negative control. A second round of transfection was then carried out at the time of seeding in trans-well inserts. Non-migrated or non-invaded cells remaining in the upper chamber were gently removed using cotton swabs, followed by two washes with 1X PBS. Stained trans-well membranes were acquired at 4× magnification using Leica DM IL LED Microscope (Leica). Invaded/ migrated cells were quantified by counting DAPI-stained nuclei using Fiji software (ImageJ). The migration/invasion data were normalized to the control condition, which was set to a value of 1.0.

### Total RNA (including small RNA) isolation

Total RNA was isolated using miRNeasy kit (Qiagen) according to manufacturer’s instructions. Total RNA quantification was carried out using the NanoDrop® ND-1000 spectrophotometer or Qubit RNA HS Assay Kit (Thermo Fisher Scientific).

### Quantitative real time-PCR (qRT-PCR) of miRNAs and mRNAs

For qRT-PCR of miR-379-3p, 10 ng of total RNA was reverse-transcribed using a TaqMan MicroRNA Reverse Transcription Kit (Thermo Fisher Scientific) and RT-specific primers for miRNAs (Thermo Fisher Scientific). 2.5 μL of RT product was pre-amplified for 14 cycles using the TaqMan PreAMP Master Mix and miRNA Taqman assay (#002133). The expression levels of miRNAs were normalized to the housekeeping gene U6 snRNA (#001973). For qRT-PCR of miR-92b-3p (477823_mir), miR-582-5p (478166_mir) and miR-29b-2-5p (478003_mir), 10 ng of total RNA was reverse-transcribed using a TaqMan™ Advanced miRNA cDNA Synthesis Kit (Thermo Fisher Scientific). 5 μL of RT product was pre-amplified for 14 cycles using the same kit. The expression levels of miRNAs were normalized to miR-16-5p (477860_mir). For qRT-PCR of transcripts, 1 μg of total RNA was reverse-transcribed using High-Capacity cDNA Reverse Transcription Kit (Thermo Fisher Scientific) or SuperScript VILO cDNA Synthesis Kit (Thermo Fisher Scientific) according to manufacturer instructions. cDNA was amplified with the TaqMan Gene Expression assay (NOTCH3 Hs01128537_m1, HES4 Hs00368353_g1, SGK3 Hs00993641_m1, Thermo Fisher Scientific) and QuantStudio 12k Flex thermocycler (Thermo Fisher Scientific) using the manufacturer’s recommended cycling conditions. Data were normalized using ESD1 (Hs00382667_m1), GUSB (Hs99999908_m1) and HPRT (Hs02800695_m1) as reference genes. Data normalization for both miRNAs and mRNAs were performed by using the delta–delta CT method or the calculation of the normalized Cq as we previously described [16].

### Cell transfection experiments

Reverse transfection was performed using Lipofectamine RNAiMAX (Thermo Fisher Scientific), following the manufacturer’s protocol. The following oligonucleotides were used at the indicated concentrations: NOTCH3 siRNA (5 nM; s9642, cat. 4427037, Thermo Fisher Scientific), SGK3 siRNA (5 nM, s24316, Thermo Fisher Scientific), or the corresponding negative control siRNA (5 nM; cat. 4390843, Thermo Fisher Scientific).

### Western blotting

Cells were lysed for ten minutes in a modified Laemmli sample buffer (2% SDS, 20% glycerol, and 125 mM Tris–HCl, pH 6.8). Equal amounts of proteins were separated by SDS–PAGE (Mini-PROTEAN® TGX TM, BioRad) and transferred on a polyvinylidene fluoride (PVDF) membrane (Bio-Rad) using Trans-blot Turbo (Bio-Rad). PVDF membranes were incubated with primary antibodies against NOTCH3 (D11B8; cat. #5276, Cell Signaling), SGK3 (D18D1; cat. # 8156, Cell Signaling) and β-Tubulin (cat. #2146, Cell Signaling) (O/N at 4 °C) and HRP-linked secondary antibodies (1 h at RT) after 1 h of blocking at RT with 5% non-fat dry milk (NOTCH3) or with 5% BSA (SGK3) in Tris-buffered saline with 0.1% Tween-20 (TBS-T). Following incubation with the chemiluminescent substrate (Clarity™ Western ECL Substrate, Bio-Rad), specific signals were captured using the ChemiDoc XRS imaging system (Bio-Rad). Densitometric analysis of Immunoblot bands was conducted using ImageLab Software (version 5.2.1, Bio-Rad) and normalized to β-Tubulin as a loading control.

### Flow cytometry

A total of 1 × 10^5^ cells were collected, resuspended in FACS staining solution (PBS 1X with 3% FBS) and then stained with DRAQ7 (invitrogen) to identify dead cells during the analysis. FACS analysis was performed using a BD FACS CANTO Cytometer. Data were processed using Flow Jo software (v.10).

### RNA-seq

For RNA-seq analysis of MIG-, INV- and CTRL A549 cells, libraries were prepared from 500 ng of total RNA using the VAHTS Universal V10 RNA-seq Library Pep Kit for Illumina (NR606, Vazyme), VAHTS DNA Adapter Set 3 (N805, Vazyme) and sequenced on a NextSeq 500 sequencing system using a paired-end, 75-cycle strategy (Illumina Inc.). The raw data were analyzed by CLC Genomics Workbench 24 (Qiagen) RNA-seq analysis pipeline. Raw data were deposited in SRA with accession # PRJNA1310781. For RNA-seq analysis on miR-92b-3p OE cells and relative control, libraries were prepared from 250 ng of total RNA using the NEGEDIA Digital mRNA-seq clinical grade sequencing service v2.0 (Next Generation Diagnostic srl), which included library preparation, quality assessment and sequencing on a NovaSeq 6000 sequencing system using a single-end, 100-cycle strategy (Illumina Inc.). The raw data were analyzed by Next Generation Diagnostic srl proprietary NEGEDIA Digital mRNA-seq pipeline (v2.0). Raw data were deposited in SRA with accession # PRJNA1310781.

### Gene set enrichment analysis (GSEA)

GSEA (GSEA, https://www.gsea-msigdb.org/gsea/index.jsp) was performed using Signal2Noise metric, 1000 random sample sets permutation, and median gene expression values for class comparison. Significant Gene Sets were considered as those with a false-discovery rate (*q*-value) less then 25% as per default GSEA setting. For enrichment analysis, we used the gene matrices *symbols.gmt* available from MSigDB.

### Analysis of publicly available external datasets

For TCGA-LUAD dataset analysis, we selected the cohort of 510 patients with lung adenocarcinomas from the TCGA data portal (https://portal.gdc.cancer.gov/) profiled for miRNA expression. Log2 read counts were used for expression analysis. Patient follow-up information was used for survival analysis: overall survival was defined as the time from the date of tumor resection until death from any cause. Follow-up was truncated at 3 years to reduce the potential overestimation of overall mortality with respect to lung cancer–specific mortality. Cox regression model was implemented on the 501 subjects with complete follow-up information, considering the normalized log2 expression of the 15 miRNAs as continuous covariates. A continuous risk score was assigned to each patient as sum of products of predictors expression times coefficient values from fitted Cox model. All statistical analyses were performed using SAS software, version 9.4 (SAS Institute, Inc., Cary, NC) and R 3.3.1 (R Core Team, 2016) and JMP 15 (SAS). *P*-values less than 0.05 were considered statistically significant.

For GSE47056 dataset (Affymetrix Multispecies miRNA-1 Array), the series matrix of already normalized data (Log2 signal intensity) was downloaded from Gene Expression Omnibus (GEO) data portal.

### Spatial transcriptomics sequencing and analysis

For each FFPE sample, FASTQ files and aligned histology images were analyzed with Space Ranger v3.3.0 using probesets annotation provided by 10X genomics (Visium Human Transcriptome Probe Set v1.0 GRCh38-2020-A). An average of 5667 median genes per spot was measured, with median genes per spot ranging from 4964 to 6672. Visium pre-processed data were then imported into R using Giotto (v 1.1.2). Spatial spots with less than 100 genes were filtered out and genes with counts in less than 50 spatial spots were discarded. Raw counts were normalized with the *normalizeGiotto* function of Giotto using the ‘scalefactor = 6000’ parameter. According to the pathologist annotation, spots were then classified as normal, tumor, necrosis, tumor infiltrating lymphocytes (TIL) and fold/artifact using Loupe Browser (10× Genomics). For each sample, tumor spots were classified as high- or low- miR-92b-3p using the PAGE function of Giotto following these steps: i) we run PAGE using genes significantly upregulated in the experiment of miR-92b-3p OE as custom Geneset; ii) the obtained enrichment score was used to classify the spots using median as cut-off. The PAGE function of Giotto was also used to calculate the enrichment score for the signature related to NOTCH1 targets derived from Molecular Signature Database (NGUYEN_NOTCH1_TARGETS_UP.v2024.1.Hs.tsv).

### Statistics

All graphs and statistical analyses were performed using Prism (version 10.2.1), SPSS (version 15.0), SAS software and R 3.3.1 (R Core Team, 2016). The details about statistical tests, number of independent replicates (N) and definition of error bars were specified in the figure legends. Exact *P*-values reported in the figures have been rounded to three decimal places. Statistical significance of migration and invasion assay results was evaluated using a two-tailed one-sample *t*-test, comparing normalized values from miR-92b-3p overexpressing cells to the mean of 1.0, corresponding to the control condition. Hierarchical clustering was performed using Cluster 3.0 (C Clustering Library 1.56; http://bonsai.hgc.jp/~mdehoon/software/cluster/software.htm) and Java Tree View (Version 1.1.6r4; http://jtreeview.sourceforge.net). The uncentered correlation and centroid linkage clustering method was used. Heatmaps were obtained by using MORPHEUS (https://software.broadinstitute.org/morpheus/) or Java Tree View. MicroRNA targets were predicted by MultiMiR R package [17].

## Results

### Lentiviral miRNA library screening identifies distinct miRNA signatures driving lung cancer proliferation, migration, and invasion

**Fig. 1:**
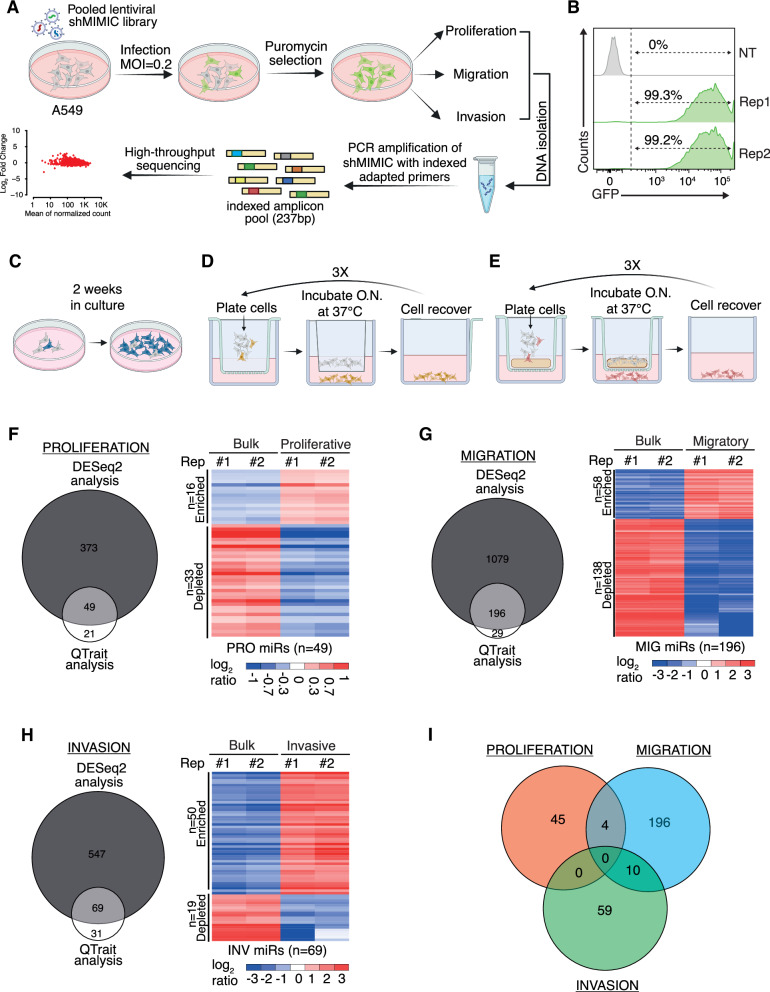
Lentiviral miRNA library screening identifies miRNAs involved in proliferation, migration and invasion of NSCLC cells. **A** Schematic workflow of lentiviral miRNA screening. **B** Flow cytometry histogram plots showing TurboGFP fluorescence intensity in A549 cells, either not-transduced (NT) or transduced with shMIMIC library and selected with puromycin. The percentage of TurboGFP-positive cells is also indicated. Schematic representation of phenotypic selection strategy to identify miRNAs associated with proliferation (**C**), migration (**D**) and invasion (**E**). (F-H) Left panels: Venn diagrams representing the overlap of miRNAs identified by both DeSeq2 Analysis and QTrait analysis of the proliferation assay (**F**), migration assay (**G**) and invasion assay (**H**). Right panels: Heatmaps showing the relative abundance of overlapping miRNAs identified in the proliferation assay (**F**), migration assay (**G**) and invasion assay (**H**). Data are log2-ratio. **I** Venn diagram representing the overlap of miRNAs associated with proliferation, migration and invasion assay.

To identify miRNAs (miRs) functionally involved in LC phenotypes of aggressiveness, we utilized a lentiviral human miRNA pooled library (shMIMICs) (Dharmacon^TM^) to overexpress mimics for virtually all human miRNome (*N* = 2580, miRbase annotation v21) (See Methods). Lung adenocarcinoma A549 cells were first transduced with shMIMICs library and subsequently treated with puromycin to eliminate non-transduced cells (Fig. 1A; see Methods). shMIMIC-A549 were then analyzed by flow cytometry to check percentage of GFP positive cells (Fig. 1B). Next, shMIMIC-A549 cells were continuously cultured in vitro for two weeks to investigate miRNAs functionally associated with LC proliferation (Fig. 1C). Alternatively, cells underwent three rounds of Transwell assays migration and invasion to derive sublines enriched in migratory or invasive phenotypes, enabling the selection of miRNAs involved in these processes (Fig. 1D, E).

We then performed NGS analysis of isolated gDNA from proliferative/migratory/invasive cells and respective control (i.e., bulk cell population) to quantify the relative abundance of miRNA mimics (Fig. 1A). To reliable score enriched/depleted miRNAs in these LC phenotypes, we used two alternative statistical approaches based on DESeq2 and Quantitative Trait Analysis (QTrait) (see Methods). In the ‘proliferation’ assay (PRO), we found 422 miRs scored by DESeq2 and 70 miRs by QTrait, with 49 miRs common to both analyses (Fig. 1F; Supplementary Table S2A). In the ‘migration’ assay (MIG), we found 1275 miRs by DESeq2 and 225 by QTrait, among which 196 miRs were shared (Fig. 1G; Supplementary Table S2B). Lastly, the ‘invasion’ assay (INV) yielded 616 miRs by DESeq2 and 100 miRs by QTrait, with 69 miRs in common (Fig. 1H; Supplementary Table S2C). Interestingly, when we compared the PRO-/MIG-/INV-miR sets (Fig. 1I) the overlap was marginal thus suggesting a certain specificity of miRNA functions.

### Identification of a ‘functional’ 15-miRNA signature predicting lung cancer progression and prognosis

**Fig. 2:**
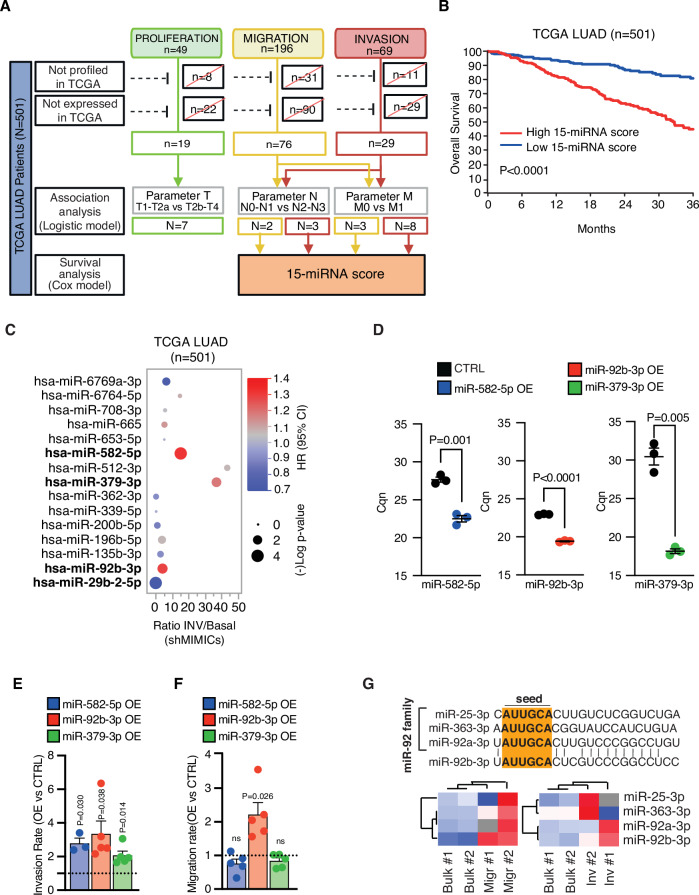
Prognostic significance and functional validation of miRNAs identified through lentiviral miRNA library screening. **A** Flow chart illustrating the analytical workflow used to evaluate the prognostic role of miRNAs identified through the lentiviral library screening using data from the TCGA-LUAD cohort. **B** Kaplan–Meier curves for 3-years overall survival stratified by high and low 15-miRNA risk-score. Log-rank *p*-values are shown for high and low 15-miRNA risk-score comparison. **C** Bubble plot showing the enrichment/depletion of 15 miRNA signature in the invasion assay and the relative prognostic significance in TCGA-LUAD dataset. Color and size of the bubbles indicate the Hazard Ratio (HR) and the *P*-value (-log *P*-value), respectively. **D** qRT-PCR of the indicated miRNAs in A549 cells transduced with lentiviral vectors overexpressing individual miRNAs (miR-582-5p, miR-92b-3p, miR-379-3p) or a not-targeting scramble sequence (CTRL). Data, expressed as normalized Cq (Cqn), are mean ± SEM (*N* = 3). *P* value was calculated by unpaired *t* test with Welch’s correction. **E** Bar plot showing the invasion rate or migration rate (**F**) of A549 cells transduced with lentiviral vectors overexpressing individual miRNAs (miR-582-5p, miR-92b-3p, miR-379-3p) or a not-targeting scramble sequence (CTRL). Data represent mean ± SEM (*n* = 3–5). *P*-value was calculated by one sample *t*-test. **G** Top Panel: Sequence alignment of miR-92 family members. Seed sequence is highlighted in orange. Bottom Panel: Heatmaps showing the relative abundance of miR-92 family members in the migration and invasion assay. Data are log2-ratio.

We then sought to validate the PRO-/MIG-/INV-miR sets in the context of lung cancer progression. Indeed, lung cancer progression depends on the interplay of biomolecular mechanisms that impact cancer cell proliferation, migration, and invasion, all of which, as previously mentioned, are influenced by miRs. We thus analyzed the expression of our PRO-, MIG-, and INV-miRs in a cohort of 501 lung adenocarcinoma (LUAD) samples from The Cancer Genome Atlas (TCGA-LUAD [4];). Logistic regression analysis revealed association of PRO-miRs with tumor size (T), a parameter that is indirectly influenced by tumor proliferation, which resulted in seven PRO-miRs significantly associated with tumor size (T1–T2a vs. T2b–T4; *p*-value < 0.05, Chi-square test; Fig. 2A; Supplementary Table S3). Furthermore, we found 5-INV and 11-MIG miRs associated with tumor spreading to lymph nodes (N2-3 vs N0-1; *p*-value < 0.05, Chi-square test; Fig. 2A; Supplementary Table S3) or metastasis to distant organs (M1 vs. M0; *p*-value < 0.05, Chi-square test; Fig. 2A; Supplementary Table S3).

Prompted by such results, we analyzed whether miRs correlating with tumor spreading/metastases, i.e. MIG-/INV-miR sets, correlate with patient adverse prognosis by fitting a Cox regression model from the expression data of the 11-MIG and 5-INV miRs (1 overlapping, i.e. miR-635-5p). Table 1 reports coefficients and hazard ratios for each miRNA, both in univariate and multivariable models. Next, we generated a continuous risk score for each patient, based on 15 miRNAs expression (see Methods), and estimated a hazard ratio (HR) of 2.72 (95% CI: 2.14–3.46, *p* < 0.0001) for one unit increase in the risk score (Table 1). Figure 2B reports the Kaplan–Meier curve for overall survival, according to high and low 15-miRNA risk-score, using the median as cut-off (median = 1.74; q1 = 1.27; q3 = 2.19). The 15-miRNA continuous risk score remained a significant independent predictor of survival, after adjustment for age at diagnosis, gender, smoking status and stage, with HR = 2.48 (1.94–3.16, *p* < 0.0001) (Table 2). Subgroup analysis revealed that the 15-miRNA risk score had equal prognostic strength, both in early stage (Stage I) and advanced stages (II-IV) (*p* for interaction = 0.93) (Table 3). Notably, addition of the 7-PRO miRs to this 15-miRNA signature (i.e., 22-miRs signature) did not improve its prognostic significance (HR, 2.44; 1.94–3.08, *p* < 0.0001; Supplementary Table S4A, B). Bubble plot of the prognostic significance (HR values) of these 15 miRNAs, along with their enrichment or depletion in highly invasive/migratory cells (shMIMICs screening), revealed that miR-582-5p, miR-379-3p, and miR-92b-3p were positively and significantly associated with poor prognosis (Fig. 2C), consistent with their enrichment in highly invasive/migratory cells. In contrast, miR-29b-2-5p was negatively and significantly correlated with poor prognosis and was accordingly depleted in highly invasive/migratory cells (shMIMICs screening) (Fig. 2C).

**Table 1:** Cox proportional hazard model in TCGA LUAD cohort (*N* = 501; *N* = 135 deaths at 3 years) for the 15 miRNAs.

	Univariate analysis			Multivariable analysis (including all 15 miRNAs)		
Predictor	Coefficients	HR (95% CI)	*p*-value (Wald test)	Coefficients	HR (95% CI)	*p*-value (Wald test)
hsa-miR-362-3p	−0.0928	0.91 (0.78–1.06)	0.2376	−0.20796	0.81 (0.68–0.97)	0.0193
hsa-miR-379-3p	0.17713	1.19 (1.05–1.35)	0.0052	0.16432	1.18 (1.01–1.38)	0.0433
hsa-miR-512-3p	0.06484	1.07 (0.97–1.17)	0.1790	0.02113	1.02 (0.91–1.15)	0.7174
hsa-miR-653-5p	0.00355	1.00 (0.90–1.12)	0.9493	0.06249	1.06 (0.96–1.19)	0.2562
hsa-miR-6769a-3p	−0.29857	0.74 (0.56–0.98)	0.0350	−0.13874	0.87 (0.65–1.16)	0.3440
hsa-miR-708-3p	0.04055	1.04 (0.89–1.22)	0.6121	−0.0002458	1.00 (0.85–1.18)	0.9977
hsa-miR-135b-3p	−0.09482	0.91 (0.80–1.03)	0.1342	0.0007987	1.00 (0.87–1.15)	0.9911
hsa-miR-196b-5p	0.06946	1.07 (1.00–1.14)	0.0376	0.00274	1.00 (0.93–1.08)	0.9442
hsa-miR-200b-5p	−0.12726	0.88 (0.74–1.04)	0.1389	−0.1631	0.85 (0.72–1.01)	0.0615
hsa-miR-29b-2-5p	−0.34766	0.71 (0.60–0.84)	<0.0001	−0.29547	0.74 (0.60–0.92)	0.0073
hsa-miR-339-5p	−0.00911	0.99 (0.82–1.20)	0.9261	0.16056	1.17 (0.96–1.44)	0.1259
hsa-miR-582-5p	0.27939	1.32 (1.20–1.46)	<0.0001	0.30245	1.35 (1.21–1.51)	<0.0001
hsa-miR-665	0.1244	1.13 (0.94–1.36)	0.1908	0.08465	1.09 (0.87–1.36)	0.4625
hsa-miR-6764-5p	0.07479	1.08 (0.84–1.38)	0.5564	0.06879	1.07 (0.83–1.39)	0.6038
hsa-miR-92b-3p	0.25722	1.29 (1.09–1.53)	0.0032	0.21842	1.24 (1.03–1.50)	0.0234
15-miRNA score	1.00001	2.72 (2.14–3.46)	<0.0001	–	–	–

**Table 2:** Multivariable Cox proportional hazard model in TCGA LUAD cohort (*N* = 491; *N* = 135 deaths at 3 years) for the 15-miRNA score, together with age at diagnosis, gender, smoking status, and stage.

Predictor	*N* (deaths)	HR (95% CI)	*p*-value (Wald test)
15-miRNA score	491 (135)	2.48 (1.94–3.16)	<0.0001
Age at diagnosis (5-unit increase)^a^	491 (135)	1.02 (1.00–1.03)	0.09
Male (vs female)	227 (66)	1.10 (0.78–1.54)	0.60
Smokers (vs never smokers)	348 (96)	1.28 (0.72–2.30)	0.40
Stage II–IV (vs stage I)	223 (95)	2.85 (1.96–4.16)	<0.0001

^a^10 individuals with missing information on age at diagnosis were excluded from the analysis, resulting in *N* = 491 individuals.

**Table 3:** Subgroup Cox proportional hazard model in TCGA LUAD cohort (*N* = 490; *N* = 135 deaths at 3 years) for the 15-miRNA score according to stage.

	15-miRNA score			
Stage^a^	*N* (*n* deaths)	HR (95% CI)	Wald test *p*-value	Interaction *p*-value
I	267 (40)	2.47 (1.52–4.00)	0.0003	0.92
II–IV	223 (95)	2.47 (1.86–3.28)	<0.0001	

Models were adjusted for age at diagnosis, gender, and smoking status.

^a^10 individuals with missing information on age at diagnosis and 1 individual with missing information on stage were excluded from the analysis, resulting in *N* = 490 individuals

### miR-92b-3p – a member of miR-92 family – drive lung adenocarcinoma invasion via NOTCH signaling

The augmented expression of miR-582-5p, miR-379-3p, and miR-92b-3p in LUAD may therefore contribute to a more invasive and metastatic phenotype of lung adenocarcinoma cells. To confirm this, we individually overexpressed (OE) miR-582-5p/379-3p/92b-3p in A549 lung adenocarcinoma cell line (Fig. 2D) and measure the rate of individual migration and invasion by using Transwell assay (see Methods). The OE of these three microRNAs significantly enhanced the invasion of A549 cells (>2 fold; *p*-value ≤ 0.05; one sample *t*-test) (Fig. 2E; Supplementary Fig. 1A–D) while only the OE of miR-92b-3p increased also cell migration (>2 fold; *p*-value ≤ 0.05; one sample *t*-test) (Fig. 2F; Supplementary Fig. 1A–D). Conversely, except for miR-582-5p whose overexpression caused a modest reduction in proliferation at the 24- and 72-hour time points (Supplemental Fig. 2A), the other investigated miRs showed no effect on cell proliferation (Supplemental Fig. 2B, C).

Intriguingly, miR-92b-3p belongs to the miR-92 family (Fig. 2G), which composes the miR-17~92 cluster, an established oncogenic miR-cluster involved in tumor progression [18]. Notably, miR-92b-3p OE increased migration and invasion in another LUAD cell line with a different genetic background, i.e. the NCI-H1975 EGFR^mut^ (L858R/T790M) cells (Supplementary Fig. 3A–C).

Strikingly, we found that all members of miR-92 family showed a trend of enrichment in invasive/migrating A549 cells (Fig. 2G). Interestingly, similar to the well-characterized miR-92a-3p [19], miR-92b-3p was also prognostic in LUAD patients from the TCGA-LUAD dataset (Supplementary Fig. 4A, B; Table 4). Notably, we confirmed the increased expression of miR-92b-3p using an external miRNA expression dataset of highly invasive A549 cells (Supplementary Fig. 5).

**Table 4:** Overall and subgroup (according to stage) Cox proportional hazard model in TCGA LUAD cohort for miR-92 family.

	Overall *N* = 491 (*n* deaths 135)		Stage I *N* = 267 (*n* deaths 40)		Stage II–IV *N* = 223 (*n* deaths 95)		Interaction with stage
	HR (95% CI)	Wald test *p*-value	HR (95% CI)	Wald test *p*-value	HR (95% CI)	Wald test *p*-value	*p*-value
hsa-miR-92b-3p	1.27 (1.07–1.50)	0.0065	1.09 (0.80–1.49)	0.60	1.32 (1.08–1.61)	0.0062	0.22
hsa-miR-92a-3p	1.23 (0.99–1.53)	0.07	0.95 (0.65–1.39)	0.80	1.37 (1.04–1.80)	0.0263	0.06
hsa-miR-25-3p	1.22 (0.95–1.57)	0.12	1.22 (0.76–1.97)	0.42	1.17 (0.87–1.58)	0.31	1.00
hsa-miR-363-3p	1.04 (0.94–1.15)	0.49	1.10 (0.89–1.37)	0.39	1.02 (0.90–1.15)	0.80	0.43

Models were adjusted for stage (overall analysis only), age at diagnosis, gender, and smoking status.

^a^10 individuals with missing information on age at diagnosis, resulting in *N* = 491 individuals; 1 additional individual with missing information on stage was excluded from the subgroup analysis.

**Fig. 3:**
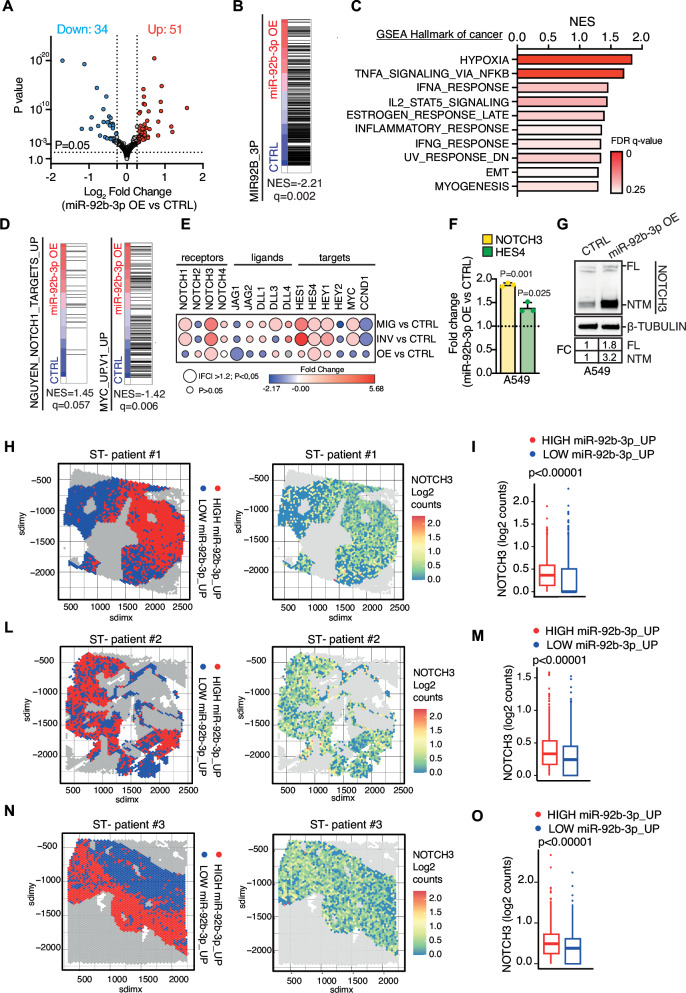
Transcriptomic and spatial analysis reveals miR-92b-3p–mediated regulation of the NOTCH pathway in LUAD cells and patient tumors. **A** Volcano plot showing differentially expressed genes in A549 overexpressing miR-92b-3p vs. CTRL. Gray dot, unchanged; blue dot, downregulated (*p* < 0.05); red dot, upregulated (*p* < 0.05); statistical significance was calculated using the Wald Test. **B** GSEA of miR-92b-3p gene set in A549 miR-92b-3p OE vs CTRL. **C** Bar plot showing GSEA results using the ‘Hallmark gene sets’ collection A549 miR-92b-3p OE vs CTRL. **D** GSEA of genes up-regulated by expression of active NOTCH1 (NGUYEN_NOTCH1_TARGETS_UP gene set) or genes up-regulated in cells overexpressing MYC (MYC_UP.V1_UP gene set) in A549 miR-92b-3p OE vs CTRL. **E** Bubble plot showing fold change expression of receptors, ligands and target of NOTCH pathway in migratory-library vs CTRL, invasion-library vs CTRL and miR-92b-3p OE vs CTRL cells. Color and size of the bubbles indicate the Fold change and the *P*-value (-log *P*-value), respectively. **F** qRT–PCR analysis of NOTCH3 and HES4 in A549 miR-92b-3p and A549 CTRL. qRT–PCR data represent the mean ± SEM (*n* = 3). Fold changes in gene expression in A549 miR-92b-3p OE cells versus CTRL cells. P-value was computed by using one sample *T*-test. **G** Immunoblot analysis of NOTCH3 in A549 miR-92b-3p OE cells versus CTRL. β-Tubulin was used as loading control. FL full length NOTCH3, NTM NOTCH3 transmembrane fragment. Results are representative of five independent observations. Left panel: Spatial distribution of tumor spots of patient #1 (**H**), patient #2 (**L**) and patient #3 (**N**) categorized either as HIGH miR-92b-3p_UP (red) or LOW miR-92b-3p_UP (blue) based on the median the enrichment score derived from genes differentially upregulated in A549 miR-92b-3p OE versus A549 CTRL. Gray spots represent area excluded from the analysis due to classification as normal tissue, necrotic tissue, tumor-infiltrating lymphocytes (TIL) or fold/artifact. Right panel: NOTCH3 miRNA levels, expressed in log2 count, in tumor spots from patient #1 (**G**), patient #2 (**I**) and patient #3 (**M**). Box plot showing NOTCH3 expression levels (log2 counts) in HIGH miR92b-3p versus LOW miR-92b-3p tumor spots of patient #1 (**I**), patient #2 (**M**) and patient #3 (**O**). *P*-value was computed using Wilcoxon Test.

To gain mechanistic insights into miR-92b-3p regulon, we then performed RNA-seq analysis of A549 cells upon miR-92b-3p OE and mock cells as control. Volcano plot showed a total of 85 significantly regulated genes (|FC|>1.2, *p* < 0.05; Wald test) (Fig. 3A). Consistently, Gene Set Enrichment Analysis (GSEA) showed miR-92b-3p targeted gene set (see Methods) significantly and negatively enriched in miR-92b-3p OE cells (Fig. 3B). Furthermore, we found mechanisms relevant for cancer progression (e.g., hypoxia/angiogenesis, inflammatory related gene sets, EMT/myogenesis, etc.) significantly and positively enriched in miR-92b-3p OE cells (Fig. 3C).

**Fig. 4:**
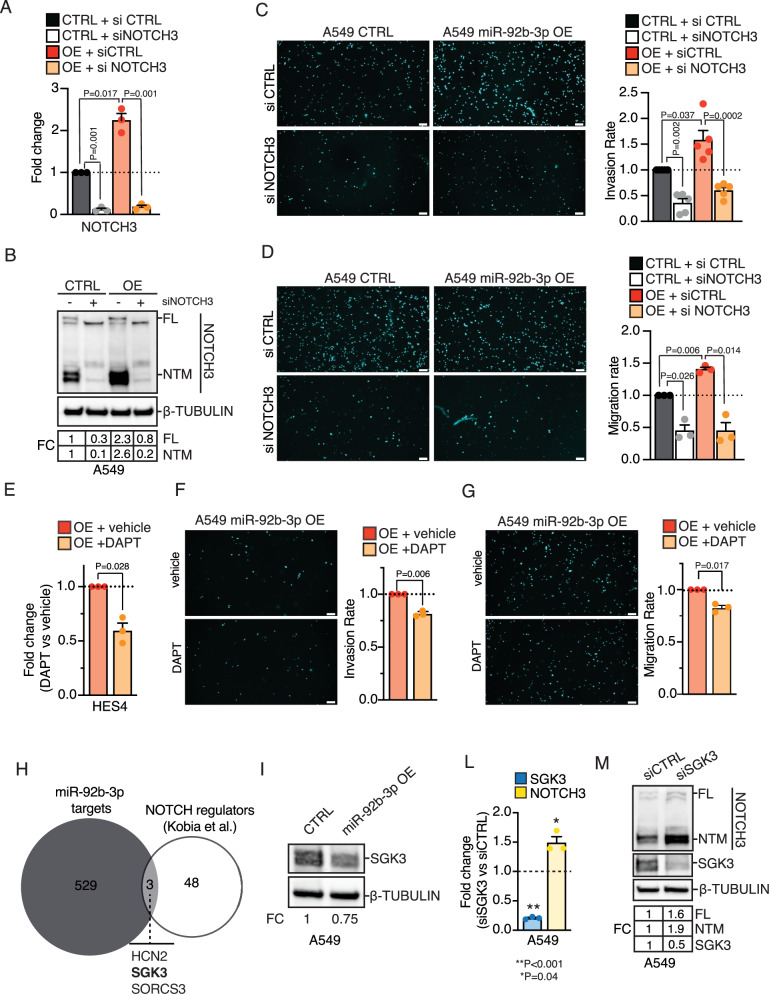
miR-92b-3p–mediated regulation of NOTCH3 modulates NSCLC cell migration and invasion. **A** qRT–PCR analysis of NOTCH3 in A549 miR-92b-3p and A549 CTRL treated with a siRNA directed to NOTCH3 (siNOTCH3) or a control sequence (siCTRL). qRT–PCR data represent the mean ± SEM (*n* = 3) and fold changes are relative to siCTRL-treated A549 CTRL cells. P-value was computed by using one sample T-test. **B** Immunoblot analysis of NOTCH3 in A549 miR-92b-3p and A549 CTRL treated with a siRNA directed to NOTCH3 (siNOTCH3) or a control sequence (siCTRL). β-Tubulin was used as loading control. FL full length, NTM NOTCH3 transmembrane fragment. Results are representative of three independent observations. **C** Left Panel: representative fluorescence image of invading A549 miR-92b-3p OE and A549 CTRL cells treated with a siRNA directed to NOTCH3 (siNOTCH3) or a control sequence (siCTRL). DAPI (light blue) visualizes nuclei. Scale Bar: 350 µm. Right panel: Bar plot showing the invasion rate of A549 miR-92b-3p and A549 CTRL cells treated with a siRNA directed to NOTCH3 (siNOTCH3) or a control sequence (siCTRL). Data represent mean ± SEM (*n* = 5). *P*-value was calculated by one sample *t*-test. **D** Left Panel: representative fluorescence image of migrating A549 miR-92b-3p OE and A549 CTRL cells treated with a siRNA directed to NOTCH3 (siNOTCH3) or a control sequence (siCTRL). DAPI (light blue) visualizes nuclei. Scale Bar: 350 µm. Right panel: Bar plot showing the migration rate of A549 miR-92b-3p and A549 CTRL cells treated with a siRNA directed to NOTCH3 (siNOTCH3) or a control sequence (siCTRL). Data represent mean ± SEM (*n* = 3). *P*-value was calculated by one sample *t*-test. **E** qRT–PCR analysis of HES4 in A549 miR-92b-3p and A549 CTRL treated with gamma-secretase inhibitor (DAPT) or vehicle solution (vehicle). qRT–PCR data represent the mean ± SEM (*n* = 3) and fold changes are relative to vehicle-treated A549 CTRL cells. *P*-value was computed by using one sample *T*-test. **F** Left Panel: representative fluorescence image of invading A549 miR-92b-3p OE treated with DAPT or vehicle solution. DAPI (light blue) visualizes nuclei. Scale Bar: 350 µm. Right panel: Bar plot showing the invasion rate of A549 miR-92b-3p OE cells treated with DAPT or vehicle solution. Data represent mean ± SEM (*n* = 3). *P*-value was calculated by one sample *t*-test. **G** Left Panel: representative fluorescence image of migrating A549 miR-92b-3p OE treated with DAPT or vehicle solution. DAPI (light blue) visualizes nuclei. Scale Bar: 350 µm. Right panel: Bar plot showing the migration rate of A549 miR-92b-3p OE cells treated with DAPT or vehicle solution. Data represent mean ± SEM (*n* = 3). *P*-value was calculated by one sample *t*-test. **H** Venn diagram of miR-92b-3p targets found by MultiMiR and the list of NOTCH regulators described by Kobia et al. **I** Immunoblot of SGK3 expression upon overexpression (OE) of miR-92b-3p in A549 cells. **L** qRT-PCR analysis of SGK3 and NOTCH3 expression in SGK3 knock-down (siSGK3) or control (siCTRL) A549 cells. **M** Immunoblot analysis of SGK3 and NOTCH3 in SGK3 knock-down (siSGK3) or control (siCTRL) A549 cells.

MiR-17~92 cluster is transcriptionally activated by lung cancer relevant oncogenes such as MYC and NOTCH1 [20, 21]. It has also been recently reported that increased miR-92b-3p expression sustain NOTCH signaling pathway and tumor progression in oral squamous carcinoma [22]. Importantly, GSEA confirmed the positive enrichment of NOTCH1 signaling (NES = 1.45; *q*-value = 0.057; Fig. 3D) and negatively enrichment of c-MYC targeted genes (NES = −1.42; *q*-value = 0.006; Fig. 3D) in miR-92b-3p OE cells, which indeed are co-regulated in a negative feedback manner by the miR-17~92 cluster [23]. Remarkably, RNA-seq analysis of MIG-/INV-A549 cells and mock cells (CTRL) transduced with shMIMICs library, revealed the increased expression of the NOTCH1 and NOTCH3 receptors, along with their target genes HEY1, HES1, and HES4, in both invasive and migratory A549 sublines (|FC|>1.2; *P* < 0.05; Fig. 3E). Consistently, miR-92b-3p OE cells exhibited elevated expression of NOTCH3 receptor and HES4 targets, as assessed by RNA-seq (Fig. 3E). Therefore, as NOTCH3 and its canonical downstream target HES4 consistently emerged as the most robustly regulated genes across all the conditions examined, we focused our validation efforts on these targets and confirmed their regulation (Fig. 3F, G) in A549 cells (KRAS^G12S^), as well as in other adenocarcinoma cell lines with different genetic backgrounds, including EGFR^T790M^ or KRAS/EGFR wild-type (Supplementary Fig. 6A–C). To validate our findings ex vivo, we performed spatial transcriptomics (ST-seq) analysis (10X Visium) on FFPE tumor sections obtained from NSCLC patients (*n* = 3; Supplementary Table S5). H&E-stained slides were reviewed, and morphological regions were annotated by a pathologist (Supplementary Fig. 7A, B). Notably, tumor spots with higher enrichment in genes upregulated by miR-92b-3p (miR-92b-3p_UP; see Methods) showed a significant upregulation of NOTCH3 expression (Fig. 3H–O). Consistently, analysis of bulk RNA sequencing data from the TCGA-LUAD cohort revealed a significant increase in NOTCH3 expression (FC = 1.17; *p* = 0.0018) in samples with high miR-92b-3p levels compared to those with low expression (Supplementary Fig. 8A). To investigate whether NOTCH3 upregulation was responsible for the increased migration/invasion observed upon miR-92-3p overexpression, we transfected A549 miR-92b-3p OE cells with siRNAs targeting NOTCH3 or negative control. Strikingly, NOTCH3 silencing (Fig. 4A, B) impaired invasion (*p* = 0.0002; Fig. 4C) and migration (*p* = 0.014; Fig. 4D) in miR-92b-3p OE cells. Similarly, when we inhibited NOTCH signaling using gamma-secretase inhibitor (DAPT) in miR-92b-3p OE cells we consistently observed a decreased expression of NOTCH target HES4 (*p* = 0.027; Fig. 4E) as well as a significant reduction in invasion (*p* = 0.006; Fig. 4F) and migration rate (*p* = 0.017; Fig. 4G). Notably, migration and invasion assays were performed at 16 h, a time point that precedes one full A549 cell doubling, which we measured at 21.1 ± 3.4 h (*N* = 8 replicates; data not shown), and consistent with previously published data [24], thereby excluding the possibility that the observed differences are attributable to altered cell viability or proliferation.

Recently, Kobia et al. performed a high-content screening to identify novel modulators of NOTCH signaling in human cancer cells [25]. We therefore leveraged this dataset by intersecting the identified NOTCH modulators with predicted miR-92b-3p targets obtained using the MultiMiR R package [17]. This analysis identified SGK3, HCN2 and SORCS3 as putative miR-92b-3p targets (Fig. 4H) and, in particular, Kobia et al. showed that SGK3 was a negative NOTCH3 regulator. Notably, SGK3 has also been reported as a validated and evolutionarily conserved target of miR-92b-3p in C2C12 murine myoblasts [26].

We then overexpressed miR-92b-3p in A549 cells and observed a significant downregulation of SGK3, as assessed by Immunoblot and qRT-PCR analyses (Fig. 4I). Importantly, SGK3 knock-down by siRNA resulted in increased NOTCH3 expression, thereby supporting a model in which miR-92b-3p positively regulates NOTCH3 through inhibition of SGK3.

## Discussion

Through the use of an shMIMIC-based functional approach we investigated the role of microRNAs in favoring lung cancer progression and found a 15-miRNA prognostic signature which was independent to other clinical and pathological factors in lung cancer patients. While this 15-miRNA signature will require further validation in independent retrospective and prospective cohorts of patients to assess its clinical utility as a prognostic biomarker, to our knowledge it represents the first miRNA prognostic signature functionally related to LC migration and invasion, and correlating to metastatic spreading to lymphnodes (i.e., the 11-MIG miRs) and distant metastases (i.e. the 5-INV miRs). Further investigations of these 15-miR signature revealed that a subset of four miRNAs showed a consistent pattern of regulation in both our shMIMIC screening and the prognostic outcomes of LUAD patients we analyzed. Intriguingly, among this 4-miRNA subset, we identified miR-92b-3p, an understudied member of the miR-17~92 cluster, which has been extensively characterized as oncogenic across multiple tumor types and associated with poor prognosis [18]. Interestingly, the same trend of enrichment was also observed for additional miR-92 family members, comprised within the miR-17~92 cluster, further reinforcing the importance of miR-92b-3p regulatory networks in the context of migratory/invasive phenotype. Notably, we have recently shown that the miR-17~92 cluster is released via extracellular vesicles (EV) in NOTCH1-driven T-ALL, functionally linked to NOTCH signaling pathways and associated with aggressive disease [27]. Coherently, we showed here by transcriptomic analysis that miR-92b-3p enhances NOTCH signaling, specifically via upregulation of NOTCH3 and its downstream target HES4. In addition, spatial transcriptomics, using ex-vivo LUAD samples, showed a significant association of high miR-92b-3p activity and increased NOTCH3 expression in tumor regions, suggesting that the miR-92b-3p/NOTCH3 axis is functionally active in vivo. Mechanistically, we found that miR-92b-3p targets SGK3 (serum/glucocorticoid-regulated kinase 3), which has been reported to negatively regulate both NOTCH1 and NOTCH3 signaling, possibly by promoting their proteasomal degradation [25].

NOTCH3 was recently described to associate with poor outcome in a multitude of tumor types including head and neck cancer (HSNCC) [22], osteosarcoma, neuroblastoma, colorectal cancer [28], breast cancer [29], pancreatic cancer [30], as well as to interact with stem-cell related mechanisms (e.g. WNT/*β*-catenin [31]), EMT [32] and angiogenesis [33, 34]. Accordingly, we demonstrated that both genetic and pharmacological inhibition of NOTCH3 suppresses migration and invasion in miR-92b-3p overexpressing cells in vitro, underscoring NOTCH3 signaling as a key driver of aggressive lung cancer phenotypes and a critical downstream effector of miR-92b-3p. Although mutations in NOTCH3 are relatively rare in lung cancer, occurring in ~4% of patients, functional studies suggest a broader relevance of its signaling activity in the context of metastatic process. NOTCH3 has been reported to associate with β-catenin in EGFR-TKI–treated non-small cell lung cancer cells, thereby increasing β-catenin stability and transcriptional activity and ultimately driving drug resistance [9, 10]. Intriguingly, tumor-associated neutrophils (TANs) upregulate NOTCH3 in cancer cells, thereby promoting NSCLC cell migration and invasion in vitro [35]. In another study, activation of NOTCH3 signaling in cancer-associated fibroblasts (CAF) lead to increased collagen production and enhances cell invasion in lung adenocarcinoma [11]. An intriguing possibility is that miR-92b-3p, beyond its tumor-intrinsic role, may also act non–cell-autonomously by activating NOTCH3 signaling in neighboring mesenchymal cells, thereby fostering a pro-metastatic microenvironment.

Importantly, although we performed shMIMICs screening in A549 cells, a widely used LUAD cell line model with KRAS G12S mutation (present in ~30–35% of LUAD patients in Western countries) [36], we also employed NCI-H1975 cells harboring the other most common EGFR alterations in LUAD, namely the L858R/T790M double mutation (present in 10–20% of LUAD patients in the Western countries [37]), and NCI-H838 cells, which KRAS/EGFR wild-type to validate the pro-invasive and migratory role of miR-92b-3p across distinct genetic backgrounds.

Despite the strengths of our findings, our study has certain limitations. First, the integration with the TCGA-LUAD dataset provided valuable clinical and transcriptomics insights, however not all miRNAs identified in our functional screen were represented thus limiting comprehensive validation across a large patient cohort. Second, ST-seq analysis was performed on a limited set of samples (*n* = 3). While the data were highly consistent with in vitro and in silico data (TCGA dataset), future studies with expanded cohorts will be important to further substantiate these findings. Third, the precise molecular mechanism by which miR-92b-3p induces NOTCH3 upregulation, as well as how NOTCH3 regulates the migratory and invasive phenotype of miR-92b-3p–overexpressing cells, remains unclear and is currently under investigation. Recently, a work by Piao et al. has reported that miR-92b-3p promotes metastasis and cisplatin resistance in oral squamous cell (OSCC) via activation the NOTCH signaling [22], specifically through the downregulation of ATXN1 and CPEB3 [22]. However, in our study, neither the ATXN1 nor the CPEB3 expressions were found modulated upon miR-92b-3p OE in vitro (Supplementary Fig. 8B). It is noteworthy that ATXN1 and CPEB3, previously reported as direct targets of miR-92b-3p in oral squamous cell carcinoma (Piao et al.), were not modulated in our lung cancer model. This discrepancy likely reflects tissue-specific stoichiometry effects of miRNA on mRNA targets and vice versa. Indeed, the activity of a given miRNA is shaped not only by its sequence complementarity but also by the cellular context, including target gene abundance, chromatin accessibility, competing endogenous RNAs, and the presence of RNA-binding proteins that modulate target recognition [38].

Future efforts will be also focused on validating the clinical utility of the 15-miRNA signature in larger prospective studies as well as using in vivo models to explore the therapeutic potential of targeting this miR-92b-3p/NOTCH3 axis to reduce lung cancer progression.

## Supplementary information

Supplemental Material

Supplemental Figures

Supplemental Table 1

Supplemental Table 2

Supplemental Table 3

Supplemental Table 4

Supplemental Table 5

Original Data WB

Original Data qRT-PCR

## Data Availability

Gene expression data generated in this study including ST-seq data are available in SRA data portal (https://www.ncbi.nlm.nih.gov/sra; PRJNA1310781). Data can be accessed by the reviewers following this link: https://dataview.ncbi.nlm.nih.gov/object/PRJNA1310781?reviewer=dsh4o9scp7bnoht4re8q1jbm94.

## References

[1] Mascaux C, Sen T, Sanchez-Cespedes M, Ortiz-Cuaran S, Bossé Y, Dammeijer F et al. Advances in lung cancer basic and translational research in 2025 – overview and perspectives focusing on NSCLC. J Thoracic Oncol. 2025;20:1369–91. 10.1016/j.jtho.2025.05.024 PMC12216557 40473107

[2] Skoulidis F, Heymach JV. Co-occurring genomic alterations in non-small-cell lung cancer biology and therapy. Nat Rev Cancer. 2019;19:495–509. 31406302 10.1038/s41568-019-0179-8 PMC7043073

[3] Wu F, Fan J, He Y, Xiong A, Yu J, Li Y, et al. Single-cell profiling of tumor heterogeneity and the microenvironment in advanced non-small cell lung cancer. Nat Commun. 2021;12:2540. 33953163 10.1038/s41467-021-22801-0 PMC8100173

[4] Cancer Genome Atlas Research Network. Comprehensive molecular profiling of lung adenocarcinoma. Nature. 2014;511:543–50. 25079552 10.1038/nature13385 PMC4231481

[5] Siegel RL, Giaquinto AN, Jemal A. Cancer statistics, 2024. CA A Cancer J Clin. 2024;74:12–49. 10.3322/caac.21820 38230766

[6] Boumelha J, De Castro A, Bah N, Cha H, De Carné Trécesson S, Rana S, et al. CRISPR–Cas9 screening identifies KRAS-induced COX2 as a driver of immunotherapy resistance in lung cancer. Cancer Res. 2024;84:2231–46. 38635884 10.1158/0008-5472.CAN-23-2627 PMC11247323

[7] Zhang Y, Donaher JL, Das S, Li X, Reinhardt F, Krall JA, et al. Genome-wide CRISPR screen identifies PRC2 and KMT2D-COMPASS as regulators of distinct EMT trajectories that contribute differentially to metastasis. Nat Cell Biol. 2022;24:554–64. 35411083 10.1038/s41556-022-00877-0 PMC9037576

[8] Peng Y, Croce CM. The role of MicroRNAs in human cancer. Sig Transduct Target Ther. 2016;1:15004. 10.1038/sigtrans.2015.4 PMC5661652 29263891

[9] Arasada RR, Shilo K, Yamada T, Zhang J, Yano S, Ghanem R, et al. Notch3-dependent β-catenin signaling mediates EGFR TKI drug persistence in EGFR mutant NSCLC. Nat Commun. 2018;9:3198. 30097569 10.1038/s41467-018-05626-2 PMC6090531

[10] Arasada RR, Amann JM, Rahman MA, Huppert SS, Carbone DP. EGFR blockade enriches for lung cancer stem–like cells through Notch3-dependent signaling. Cancer Res. 2014;74:5572–84. 25125655 10.1158/0008-5472.CAN-13-3724 PMC4263272

[11] Xiang H, Pan Y, Sze MA, Wlodarska M, Li L, Van De Mark KA, et al. Single-cell analysis identifies NOTCH3-mediated interactions between stromal cells that promote microenvironment remodeling and invasion in lung adenocarcinoma. Cancer Res. 2024;84:1410–25. 38335304 10.1158/0008-5472.CAN-23-1183 PMC11063690

[12] Cerciello F, Lane L, Bianchi F, Zhang J, Giraldo T, Samarasinghe K, et al. PT1.03.04 VIKTOR - the virtual international knowledgebase for thoracic oncology research of the IASLC. J Thorac Oncol. 2025;20:S591.

[13] Langmead B, Trapnell C, Pop M, Salzberg SL. Ultrafast and memory-efficient alignment of short DNA sequences to the human genome. Genome Biol. 2009;10:R25. 19261174 10.1186/gb-2009-10-3-r25 PMC2690996

[14] Love MI, Huber W, Anders S. Moderated estimation of fold change and dispersion for RNA-seq data with DESeq2. Genome Biol. 2014;15:550. 25516281 10.1186/s13059-014-0550-8 PMC4302049

[15] Simon R, Lam A, Li M-C, Ngan M, Menenzes S, Zhao Y. Analysis of gene expression data using BRB-array tools. Cancer Inf. 2007;3:117693510700300. PMC2675854 19455231

[16] Montani F, Marzi MJ, Dezi F, Dama E, Carletti RM, Bonizzi G et al. miR-Test: a blood test for lung cancer early detection. J Natl Cancer Inst. 2015;107:djv063. 10.1093/jnci/djv063 25794889

[17] Ru Y, Kechris KJ, Tabakoff B, Hoffman P, Radcliffe RA, Bowler R, et al. The multiMiR R package and database: integration of microRNA–target interactions along with their disease and drug associations. Nucleic Acids Res. 2014;42:e133–e133. 25063298 10.1093/nar/gku631 PMC4176155

[18] Mogilyansky E, Rigoutsos I. The miR-17/92 cluster: a comprehensive update on its genomics, genetics, functions and increasingly important and numerous roles in health and disease. Cell Death Differ. 2013;20:1603–14. 24212931 10.1038/cdd.2013.125 PMC3824591

[19] Huang Y-F, Liu M-W, Xia H-B, He R. Expression of miR-92a is associated with the prognosis in non-small cell lung cancer: An observation study. Med (Baltim). 2022;101:e30970. 10.1097/MD.0000000000030970 PMC9575788 36254053

[20] O’Donnell KA, Wentzel EA, Zeller KI, Dang CV, Mendell JT. c-Myc-regulated microRNAs modulate E2F1 expression. Nature. 2005;435:839–43. 15944709 10.1038/nature03677

[21] Yamashita AS, Geraldo MV, Fuziwara CS, Kulcsar MAV, Friguglietti CUM, Da Costa RB, et al. Notch pathway is activated by MAPK signaling and influences papillary thyroid cancer proliferation. Transl Oncol. 2013;6:197–IN22. 23544172 10.1593/tlo.12442 PMC3610552

[22] Piao Y, Jung S-N, Lim MA, Zheng S, Oh C, Jin YL, et al. The role of miR-92b-3p in notch signaling and monitoring of oral squamous cell carcinoma. Oncogene. 2025;44:1300–11. 39966557 10.1038/s41388-025-03306-2

[23] Mihailovich M, Bremang M, Spadotto V, Musiani D, Vitale E, Varano G, et al. miR-17-92 fine-tunes MYC expression and function to ensure optimal B cell lymphoma growth. Nat Commun. 2015;6:8725. 26555894 10.1038/ncomms9725 PMC4667639

[24] Kunkel MW, Coussens NP, Morris J, Taylor RC, Dexheimer TS, Jones EM, et al. HTS384 NCI60: the next phase of the NCI60 screen. Cancer Res. 2024;84:2403–16. 38861359 10.1158/0008-5472.CAN-23-3031 PMC11292194

[25] Kobia FM, Castro E Almeida L, Paganoni AJ, Carminati F, Andronache A, Lavezzari F, et al. Novel determinants of NOTCH1 trafficking and signaling in breast epithelial cells. Life Sci Alliance. 2025;8:e202403122. 39663000 10.26508/lsa.202403122 PMC11633778

[26] Ye Z, Shi J, Ning Z, Hou L, Hu CY, Wang C. MiR-92b-3p inhibits proliferation and migration of C2C12 cells. Cell Cycle. 2020;19:2906–17. 33043788 10.1080/15384101.2020.1827511 PMC7714464

[27] Colangelo T, Panelli P, Mazzarelli F, Tamiro F, Melocchi V, De Santis E, et al. Extracellular vesicle microRNAs contribute to Notch signaling pathway in T-cell acute lymphoblastic leukemia. Mol Cancer. 2022;21:226. 36550553 10.1186/s12943-022-01698-3 PMC9773489

[28] Sugiura K, Masuike Y, Suzuki K, Shin AE, Sakai N, Matsubara H, et al. LIN28B promotes cell invasion and colorectal cancer metastasis via CLDN1 and NOTCH3. JCI Insight. 2023;8:e167310. 37318881 10.1172/jci.insight.167310 PMC10443801

[29] Leontovich AA, Jalalirad M, Salisbury JL, Mills L, Haddox C, Schroeder M, et al. NOTCH3 expression is linked to breast cancer seeding and distant metastasis. Breast Cancer Res. 2018;20:105. 30180881 10.1186/s13058-018-1020-0 PMC6123953

[30] Lin H, Hu P, Zhang H, Deng Y, Yang Z, Zhang L. GATA2-mediated transcriptional activation of Notch3 promotes pancreatic cancer liver metastasis. Mol Cells. 2022;45:329–42. 35534193 10.14348/molcells.2022.2176 PMC9095506

[31] Wang T, Holt CM, Xu C, Ridley C, P O Jones R, Baron M, et al. Notch3 activation modulates cell growth behaviour and cross-talk to Wnt/TCF signalling pathway. Cell Signal. 2007;19:2458–67. 17822871 10.1016/j.cellsig.2007.07.019

[32] Sun J, Dong M, Xiang X, Zhang S, Wen D. Notch signaling and targeted therapy in non-small cell lung cancer. Cancer Lett. 2024;585:216647. 38301911 10.1016/j.canlet.2024.216647

[33] Liu H, Zhang W, Kennard S, Caldwell RB, Lilly B. Notch3 is critical for proper angiogenesis and mural cell investment. Circ Res. 2010;107:860–70. 20689064 10.1161/CIRCRESAHA.110.218271 PMC2948576

[34] Daido W, Nakashima T, Masuda T, Sakamoto S, Yamaguchi K, Horimasu Y, et al. Nestin and Notch3 collaboratively regulate angiogenesis, collagen production, and endothelial–mesenchymal transition in lung endothelial cells. Cell Commun Signal. 2023;21:247. 37735673 10.1186/s12964-023-01099-z PMC10512559

[35] Peng W, Sheng Y, Xiao H, Ye Y, Kwantwi LB, Cheng L, et al. Lung adenocarcinoma cells promote self-migration and self-invasion by activating neutrophils to upregulate Notch3 expression of cancer cells. Front Mol Biosci. 2022;8:762729. 35118116 10.3389/fmolb.2021.762729 PMC8804382

[36] Judd J, Abdel Karim N, Khan H, Naqash AR, Baca Y, Xiu J, et al. Characterization of KRAS mutation subtypes in non–small cell lung cancer. Mol Cancer Therapeutics. 2021;20:2577–84. 10.1158/1535-7163.MCT-21-0201 PMC9662933 34518295

[37] Melosky B, Kambartel K, Häntschel M, Bennetts M, Nickens DJ, Brinkmann J, et al. Worldwide prevalence of epidermal growth factor receptor mutations in non-small cell lung cancer: a meta-analysis. Mol Diagn Ther. 2022;26:7–18. 34813053 10.1007/s40291-021-00563-1 PMC8766385

[38] Diener C, Keller A, Meese E. The miRNA–target interactions: an underestimated intricacy. Nucleic Acids Res. 2024;52:1544–57. 38033323 10.1093/nar/gkad1142 PMC10899768

