# Supplementary material for: Functional analysis of the human miRNome in non-small cell lung cancer unveils a novel miR-92b-3p/NOTCH3 axis that drives tumor progression
Source: Cell Death Dis. 2026 Apr 8;17(1):502. doi: 10.1038/s41419-026-08709-x (PMC13187004; doi:10.1038/s41419-026-08709-x)
Supplement: Supplementary file 1 — Supplemental Material [file 41419_2026_8709_MOESM1_ESM.docx]

**Functional analysis of the human miRNome in non-small cell lung cancer unveils a novel miR-92b-3p/NOTCH3 axis that drives tumor progression.**

**SUPPLEMENTAL MATERIAL**

**SUPPLEMENTAL TABLES LEGENDS**

**Table S1. Normalized counts of miRNAs in the proliferation (PRO), migration (MIG) and invasion (INV) assay.** Lists of miRNAs (with MIMAT_ID) and their normalized counts in the PRO (A), MIG (B) and INV (C) assay. DESeq2 and BRB-Qtrait p-values are reported. NS, not significant.

**Table S2. Enriched/depleted miRNAs in the proliferation (PRO), migration (MIG) and invasion (INV) assay.** Lists of miRNAs (with MIMAT IDs) associated with PRO (A), MIG (B), and INV (C). For each miRNA, fold-changes in individual replicates and the corresponding mean fold-change (Mean_FC) are shown. The direction of regulation (up/down) and concordance across replicates are indicated. DESeq2 and BRB-Qtrait p-values are reported, together with the indication of the subset of miRNAs passing predefined thresholds in both analyses.

**Table S3. Association of proliferative (PRO), migratory (MIG), and invasive (INV) miRNAs with tumor size, lymph node involvement, and metastasis (TNM parameters) in the TCGA LUAD cohort.** List of miRNAs (with MIMAT IDs) categorized as PRO-, INV-, or MIG-miRNAs, together with their observed enrichment trends (up- or downregulated). Associations were tested with tumor size (T1–T2a vs. T2b–T4), lymph node involvement (N0–N1 vs. N2–N3), and metastasis status (M0 vs. M1) using chi-square test. P-values are reported.

**Table S4. Multivariable Cox proportional hazards models in the TCGA LUAD cohort evaluating the prognostic value of 22-miRNA and 15-miRNA scores.** (A) Model including the 22-miRNA score as predictor, adjusted for age at diagnosis, gender, smoking status, and stage (TCGA LUAD, N = 491 with follow-up and clinicopathological data; 135 deaths within 3 years). (B) Model including the 15-miRNA score as predictor, adjusted for age at diagnosis, gender, smoking status, and stage (TCGA LUAD, N = 491 with follow-up and clinicopathological data; 135 deaths within 3 years).

**Table S5. Clinical-pathological feature of patients' sample used in ST.** Clinical information of patients used in spatial transcriptomics (ST). Abbreviation: WT, Wild type; NA, Not available; Neg, negative.

**SUPPLEMENTAL FIGURES LEGENDS**

**Supplemental Figure 1 – Functional validation of selected miRNAs in regulating migration and invasion of A549 cells.**

Representative fluorescence image of invading (left) and migrating (right) A549 cells transduced with lentiviral vectors overexpressing individual miRNAs (miR-582-5p, miR-92b-3p, miR-379-3p) or a not-targeting scramble sequence (CTRL). DAPI (light blue) visualizes nuclei. Scale Bar: 350µM.

**Supplemental Figure 2- Effects of selected miRNAs in regulating the proliferation of a549 cells**

(A) Proliferation rate of A549 CTRL and A549 miR-92b-3p OE cells cultured for 96 hours. Fold changes of proliferation rate are calculated relative to the time of seeding. Bars represent mean ± SEM (*n* = 3). A two‐tailed unpaired *t*‐test was performed. (B) Proliferation rate of A549 CTRL and A549 miR-582-5p OE cells cultured for 96 hours. Fold changes are calculated relative to the time of seeding. Bars represent mean ± SEM (*n* = 3). A two‐tailed unpaired *t*‐test was performed. (C) Proliferation rate of A549 CTRL and A549 miR-379-3p OE cells cultured for 96 hours. Fold changes are calculated relative to the time of seeding. Bars represent mean ± SEM (*n* = 3). A two‐tailed unpaired *t*‐test was performed.

**Supplemental Figure 3 – Role of miR-92b-3p in regulating the migration and invasion of NCI-H1975**

(A) qRT-PCR of the miR-92b-3p expression in NCI-H1975 cells transduced with lentiviral vectors overexpressing miR-92b-3p or a not-targeting scramble sequence (CTRL). Data, expressed as normalized Cq (Cqn), are mean ± SEM (N = 3). P-value was calculated by unpaired t test with Welch's correction. (B) Left panel: Representative fluorescence image of invading NCI-H1975 miR-92b-3p OE versus CTRL. DAPI (light blue) visualizes nuclei. Scale Bar: 350µM. Right panel: Bar plot showing the invasion rate of NCI-H1975 miR-92b-3p OE versus CTRL. Data represent mean ± SEM (n=4). P-value was calculated by one sample t-test. (C) Left panel: Representative fluorescence image of migrating NCI-H1975 miR-92b-3p OE versus CTRL. DAPI (light blue) visualizes nuclei. Scale Bar: 350µM. Right panel: Bar plot showing the migration rate of NCI-H1975 miR-92b-3p OE versus CTRL. Data represent mean ± SEM (n=5). P-value was calculated by one sample t-test.

**Supplemental Figure 4 – Survival analysis of TGCA-LUAD patients according miR-92b-3p and miR-92a-3p expression**

(A) Kaplan–Meier curves for 3-years overall survival of TGCA-LUAD patients stratified by high and low miR-92b-3p expression in all stages (left), only stage I (center) and advanced stages (right). Log-rank p-values are shown for high and low miR-92b-3p comparison. (B) Kaplan–Meier curves for 3-years overall survival of TGCA-LUAD patients stratified by high and low miR-92a-3p expression in all stages (left), only stage I (center) and advanced stages (right). Log-rank p-values are shown for high and low miR-92b-3p comparison.

**Supplemental Figure 5 – Analysis of the 15-miRNA signature in a publicly available dataset comparing highly invasive and low invasive A549 human lung adenocarcinoma cells**

Bubble plot showing the enrichment/depletion of 15 miRNA signature in the invasive-A549 (INV) vs. bulk cells (Basal) and the relative expression regulation in the GSE47056 dataset. Color and size of the bubbles indicate the log2 ratio and the P-value (-log P-value), respectively.

**Supplemental Figure 6 – Gene expression analysis of NOTCH3 in different experimental settings.**

(A) On the left, fold changes in NOTCH3 and HES4 expression in NCI-H1975 (EGFRmut, T790M) miR-92b-3p OE cells versus NCI-H1975 CTRL cells (see also Supplemental Figure 3A). qRT–PCR data represent the mean ± SEM (*n* = 3). P-value was computed by using one sample T-test. On the right, immunoblot analysis of NOTCH3 in NCI-H1975 miR-92b-3p OE cells versus CTRL. β-Tubulin was used as loading control. FL: full length NOTCH3; NTM: NOTCH3 transmembrane fragment. Results are representative of three independent observations. (B) On the left, qRT–PCR analysis of the miR-92b-3p expression in NCI-H1944 cells (KRASmut, G13N) transduced with lentiviral vectors overexpressing miR-92b-3p or a not-targeting scramble sequence (CTRL). Data, expressed as normalized Cq (Cqn), are mean ± SEM (N = 3). P-value was calculated by unpaired t test with Welch's correction. On the right, fold changes in NOTCH3 and HES4 expression in NCI-H1944 miR-92b-3p OE cells versus NCI-H1944 CTRL cells. qRT–PCR data represent the mean ± SEM (*n* = 3). P-value was computed by using one sample T-test. (C) On the left, qRT–PCR analysis of the miR-92b-3p expression in NCI-H838 cells (KRAS and EGFR wild-type) transduced with lentiviral vectors overexpressing miR-92b-3p or a not-targeting scramble sequence (CTRL). Data, expressed as normalized Cq (Cqn), are mean ± SEM (N = 3). P-value was calculated by unpaired t test with Welch's correction. On the right, fold changes in NOTCH3 and HES4 expression in NCI-H838 miR-92b-3p OE cells versus NCI-H838 CTRL cells. qRT–PCR data represent the mean ± SEM (*n* = 3). P-value was computed by using one sample T-test.

**Supplemental Figure 7 – Histological staining and pathologist annotations of spatial transcriptomic slides from LUAD patients.**

(A) Hematoxylin and eosin (H&E) staining of tumor tissues on the 10x Visium capture slides from patient#1 (top), patient#2 (center) and patient#3 (bottom). (B) Pathologist annotation of spots captured with 10x Visium capture slides from patient#1 (top), patient#2 (center) and patient#3 (bottom).

**Supplemental Figure 8**

(A) Box plot analysis showing the expression of NOTCH3 gene in the TCGA-LUAD cohort of samples. Y-axes, Log2 normalized counts; X-axes, samples categories defined by the median of expression of miR-92b-3p in the samples of the cohort (i.e. High- miR-92b-3p ≥ median; Low- < median). P-value was calculated using Wald Test. (B) ATXN1 and CPEB3 expression profile analysis (RNA-seq) upon miR-92b-3p OE in A549 cells and A549 CTRL cells. Y-axes, Log2 normalized counts; X-axes, samples categories as per the legend. P-value was calculated using Wald Test.
