# Supplementary material for: Functional analysis of the human miRNome in non-small cell lung cancer unveils a novel miR-92b-3p/NOTCH3 axis that drives tumor progression
Source: Cell Death Dis. 2026 Apr 8;17(1):502. doi: 10.1038/s41419-026-08709-x (PMC13187004; doi:10.1038/s41419-026-08709-x)

Supplemental Figure 1

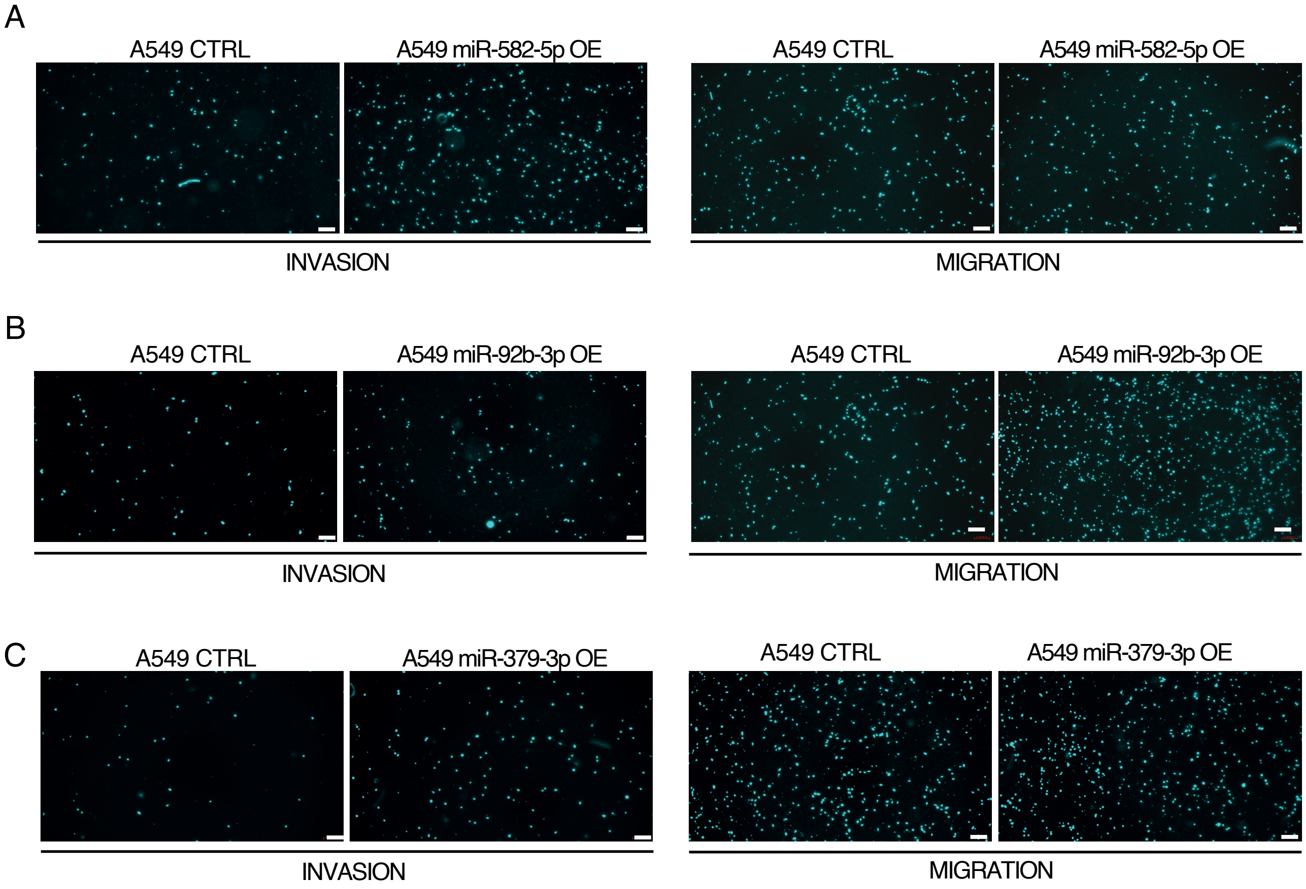

Supplemental Figure 2

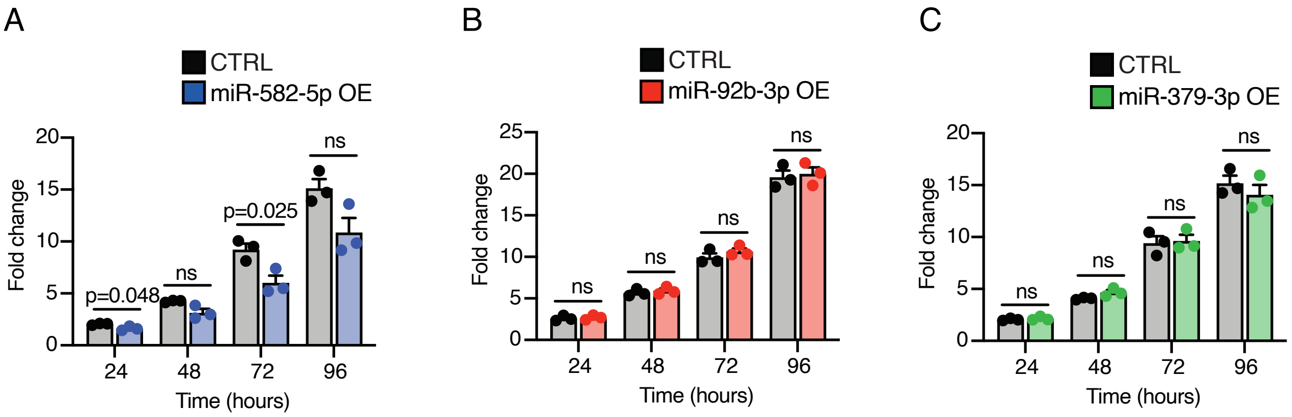

Supplemental Figure 3

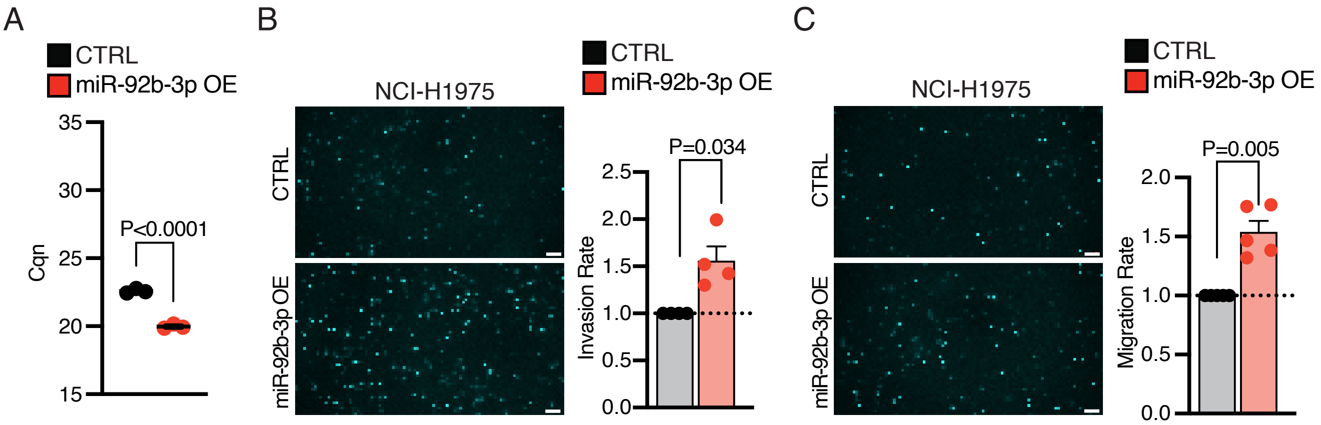

Supplemental Figure 4

A

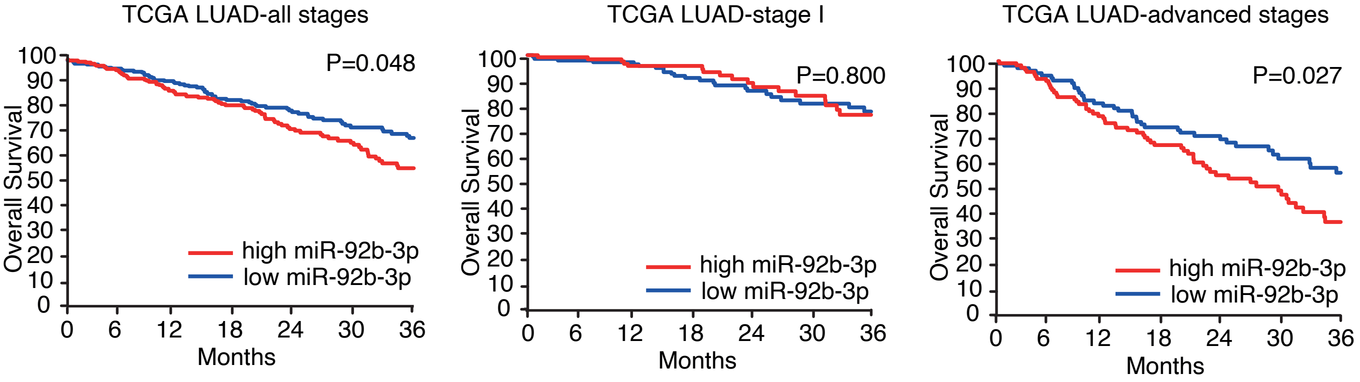

B

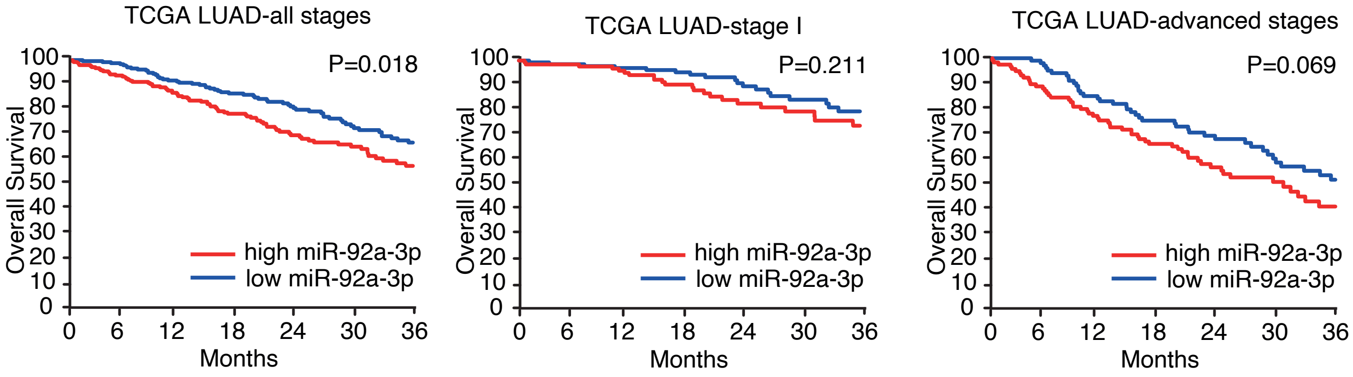

Supplemental Figure 5

A

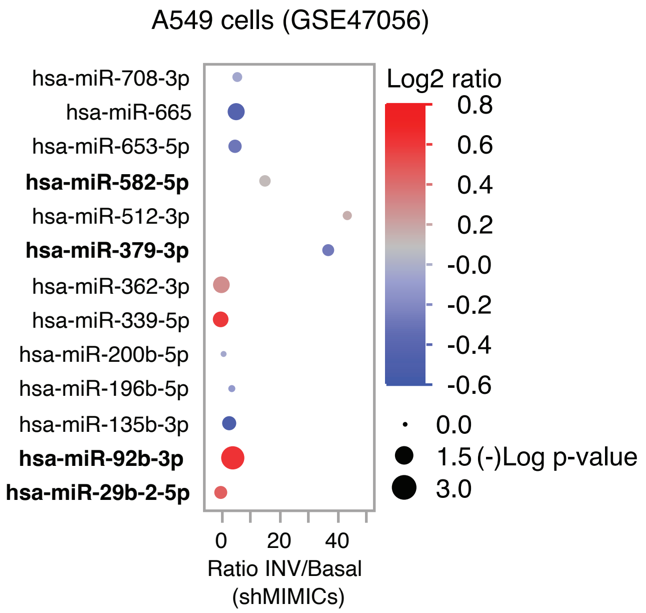

Supplemental Figure 6

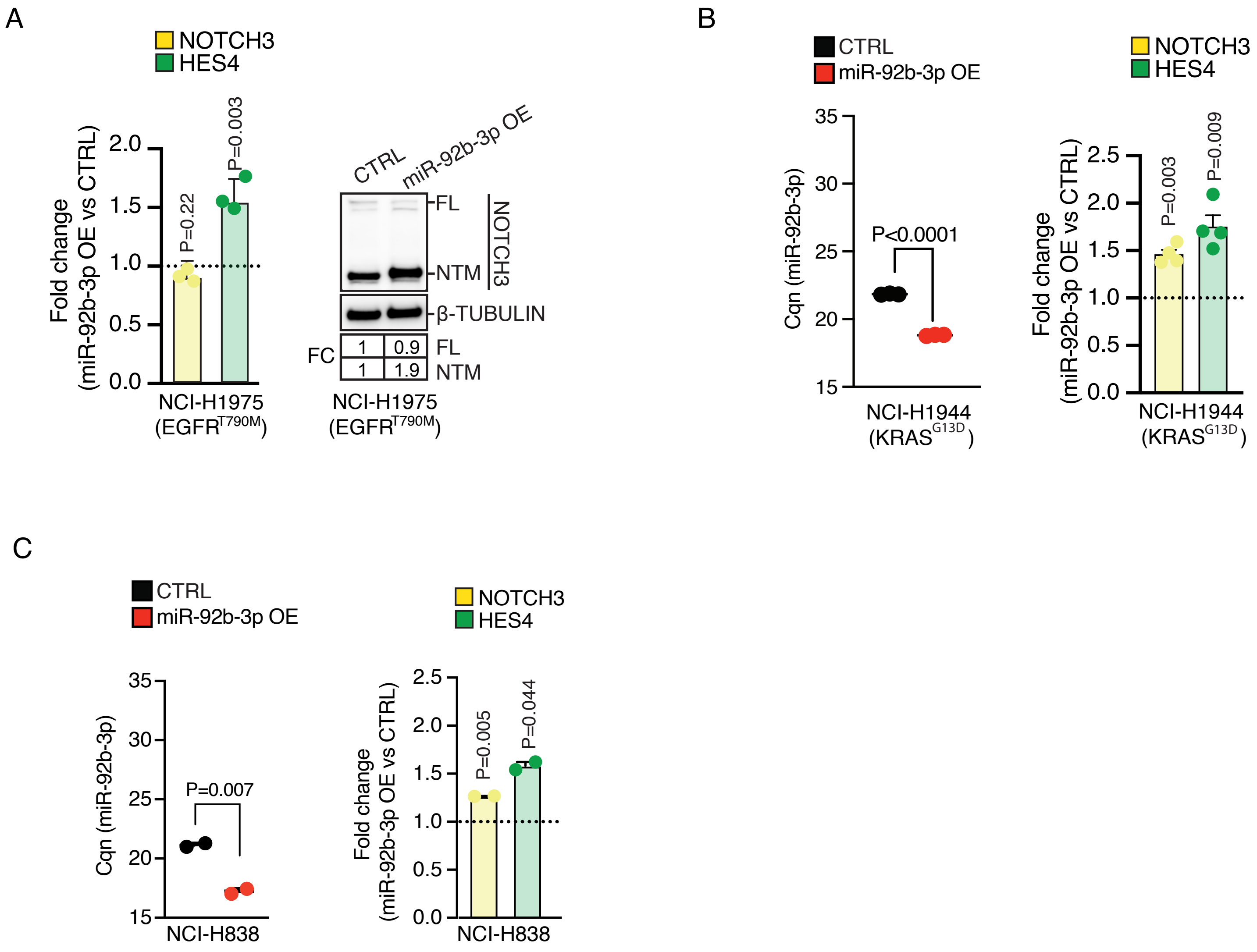

Supplemental Figure 7

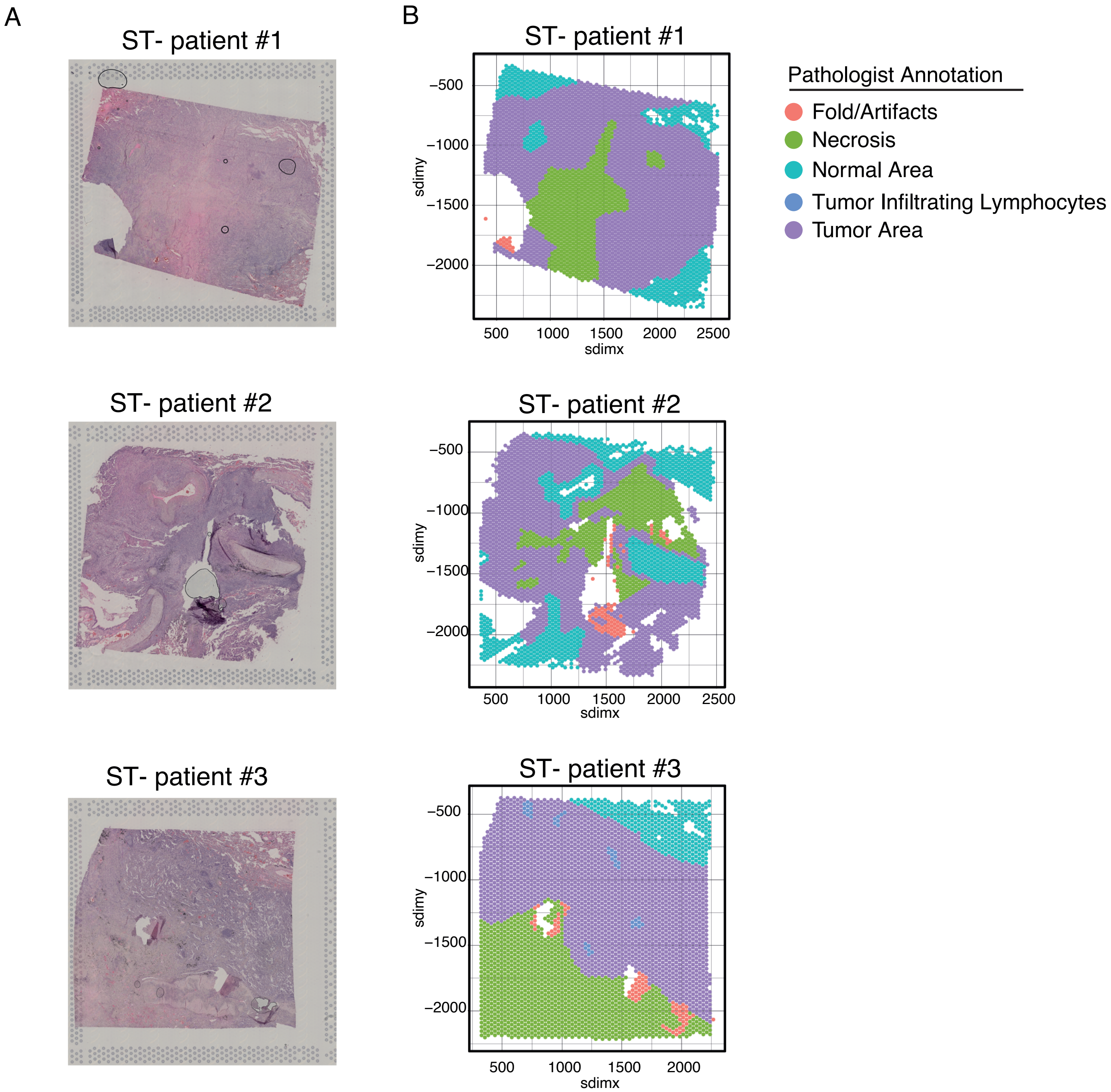

Supplemental Figure 8

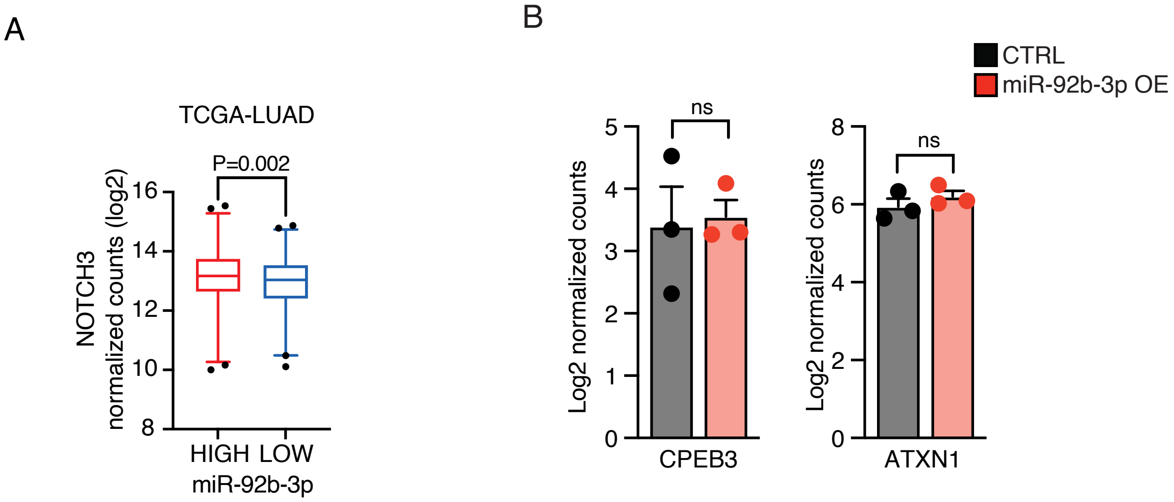

Supplement: Supplementary file 2 — Supplemental Figures [file 41419_2026_8709_MOESM2_ESM.pdf]
