# Supplementary material for: Functional analysis of the human miRNome in non-small cell lung cancer unveils a novel miR-92b-3p/NOTCH3 axis that drives tumor progression
Source: Cell Death Dis. 2026 Apr 8;17(1):502. doi: 10.1038/s41419-026-08709-x (PMC13187004; doi:10.1038/s41419-026-08709-x)

Full unedited gel for Figure 3G

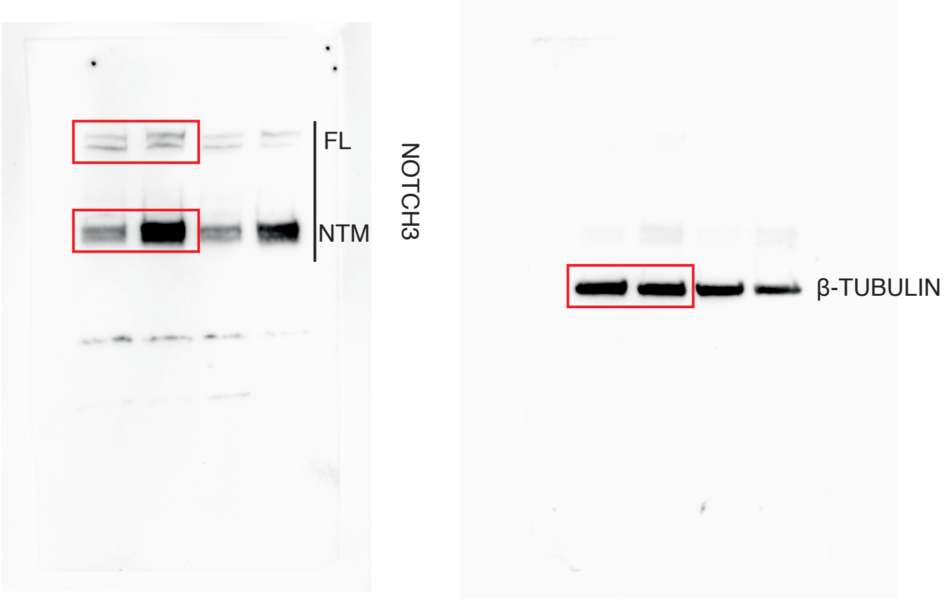

Full unedited gel for Figure 4B

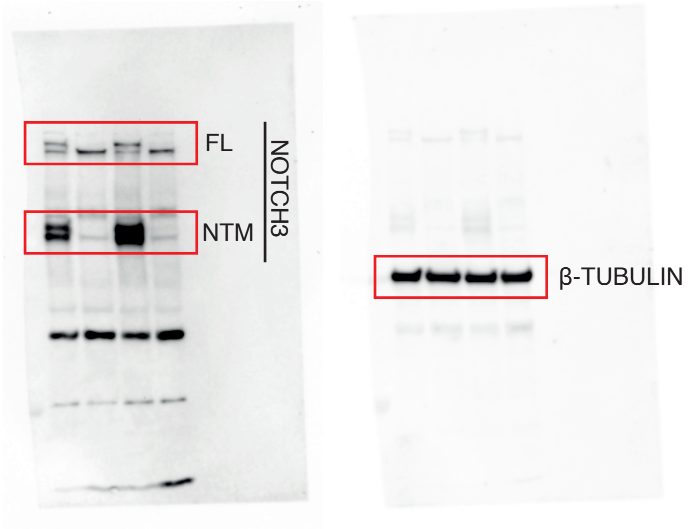

Full unedited gel for Figure 4I

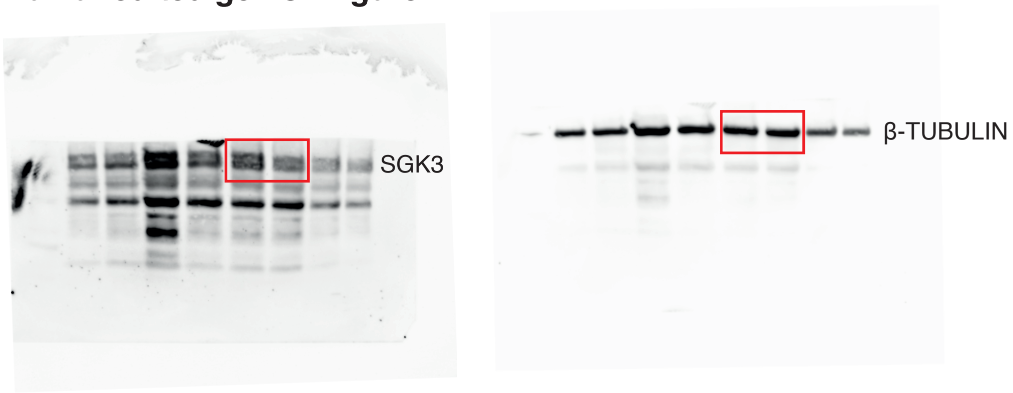

Full unedited gel for Figure 4M

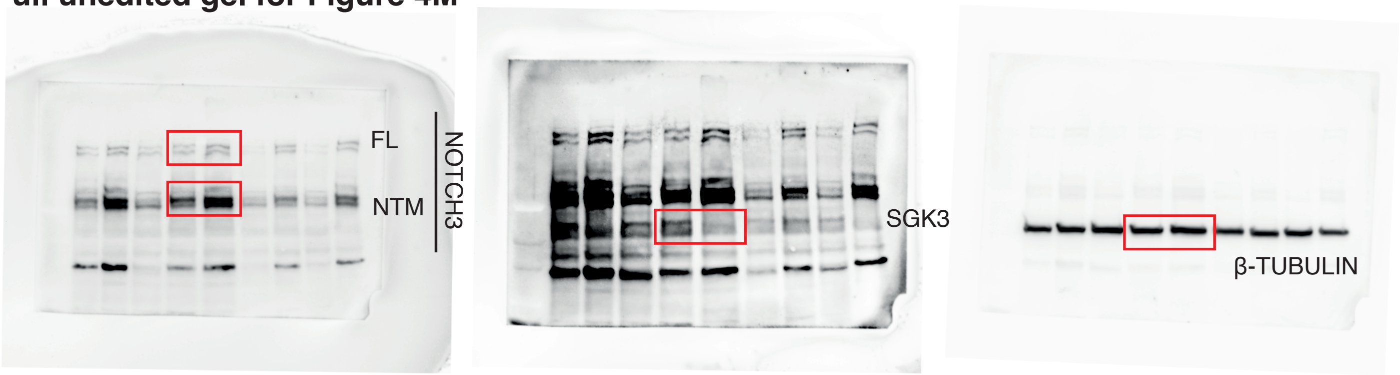

Full unedited gel for Supplemental Figure 6A

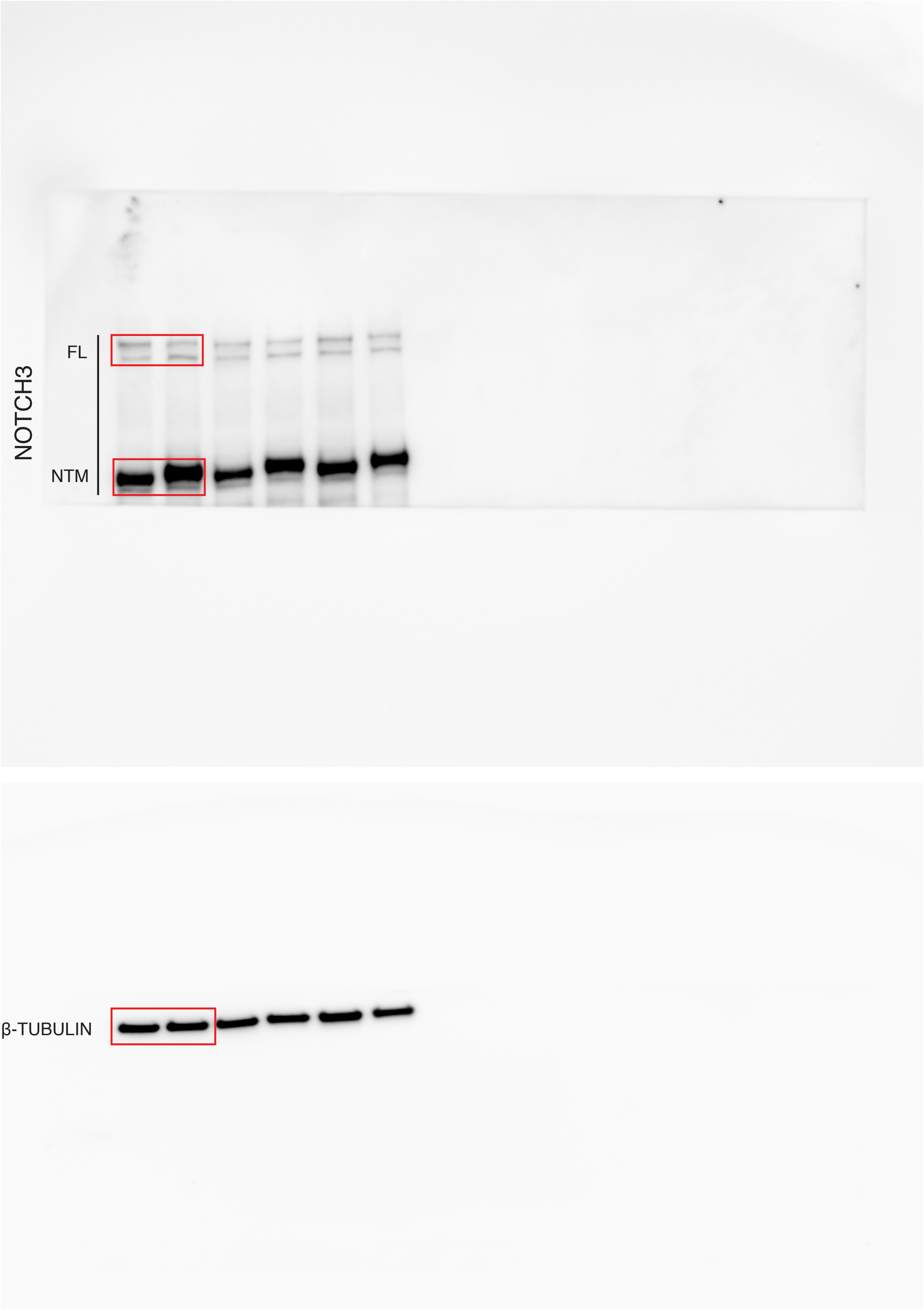

Supplement: Supplementary file 8 — Original Data WB [file 41419_2026_8709_MOESM8_ESM.pdf]
